# Peer cooperation and competition are both positively linked with mastery‐approach goals: An achievement goal perspective

**DOI:** 10.1111/bjep.12784

**Published:** 2025-06-09

**Authors:** Yikang Chen, Jiajing Li, Harold Chui, Ronnel B. King

**Affiliations:** ^1^ Department of Educational Psychology, Faculty of Education The Chinese University of Hong Kong Hong Kong SAR China; ^2^ College of Education for the Future Beijing Normal University Zhuhai China; ^3^ Department of Curriculum and Instruction, Faculty of Education The Chinese University of Hong Kong Hong Kong SAR China

**Keywords:** contexts of learning, mastery‐approach goals, motivation, peer relationships, secondary/high schools

## Abstract

**Background:**

Research on the predictors of mastery‐approach goals has focused primarily on the role of internal psychological and teacher‐related factors. However, the role of one's peers, specifically peer cooperation and competition, has seldom been explored.

**Aims:**

Peer cooperation and competition could be studied at either the individual‐ or school‐level. The present study examined whether individual‐level and school‐level peer cooperation and competition were associated with mastery‐approach goals.

**Sample:**

Data came from 565,732 students nested within 20,227 schools across 75 countries.

**Methods:**

Doubly latent multilevel structural equation modelling was used. We modelled peer cooperation and competition at both the individual and school levels.

**Results:**

In line with expectations, both individual‐level and school‐level peer cooperation were positively associated with mastery‐approach goals. Surprisingly, individual and school‐level peer competition were also positively linked with mastery‐approach goals. It seems that viewing competition as purely maladaptive might be an oversimplification, as competition among peers might also facilitate self‐improvement and mastery.

**Conclusion:**

This study highlights the importance of peers in students' pursuit of mastery‐approach goals. Furthermore, it emphasizes the importance of peer cooperation and the need for a more nuanced consideration of peer competition.

## INTRODUCTION

Mastery‐approach goals, which refer to the development of one's competence relative to self‐set standards (Elliot, 2005; Elliot & Hulleman, 2017), are considered the most optimal and beneficial type of achievement goal. They are associated with higher levels of intrinsic motivation, interest, and performance (Elliot, 1999; Elliot et al., 2011; Katz‐Vago & Benita, 2024). Given the importance of mastery‐approach goals, much work has been devoted to understanding the factors that facilitate them (Hulleman et al., 2010; Payne et al., 2007).

Prior work has focused on the role of internal psychological factors in mastery‐approach goals. For example, personality traits (e.g., conscientiousness), motives (e.g., work mastery and fear of failure), and implicit theories of intelligence or mindsets (e.g., growth versus fixed mindset) have all been found to be associated with mastery‐approach goals (Bartels & Magun‐Jackson, 2009; Dweck & Master, 2012; Elliot et al., 2010; Yu & McLellan, 2020). Research has also extensively explored the role of social contexts, with particular attention paid to teachers (Skinner, 2023; Wentzel & Skinner, 2022). For example, positive relationships with teachers have been found to be key drivers of mastery‐approach goals (e.g., Benita & Matos, 2021; Cho et al., 2011).

However, whether and how peers might also be associated with mastery‐approach goals is still relatively unexplored. This is an important gap because peers could strongly shape students' motivation (e.g., Skinner, 2023; Wentzel & Skinner, 2022). Students spend a significant amount of time with their peers and are sensitive to peer influence during this developmental period (Brown et al., 2008; Giletta et al., 2021; Scholte & Van Aken, 2020). Given the importance of peers during adolescence (Shim et al., 2013), coupled with adolescents' tendency to be influenced by peer norms (King, 2020; King & Mendoza, 2020; Skinner et al., 2022; Wang et al., 2018), it is crucial to explore how peers might play a role in students' mastery‐approach goals.

To address these gaps, the current study aims to examine the role of peers in understanding mastery‐approach goals using a cross‐cultural dataset drawing on 75 countries across the globe. We particularly focus on peer cooperation and competition as these are the primary forms of social interaction among peers (Brown et al., 2008) and have been identified as central to students' motivational outcomes (Johnson & Johnson, 2018; Richard et al., 2002).

### The achievement goal perspective

One of the most important frameworks that can be used to understand student motivation is the achievement goal perspective (Sommet & Elliot, 2020). It differentiates two primary types of goals: mastery goals and performance goals (Elliot, 1999; Elliot et al., 2011). Mastery goals emphasize personal growth, self‐improvement and the development of competence (Elliot et al., 2015). Performance goals involve demonstrating competence relative to others (Elliot, 1999; Elliot & Moller, 2003). Subsequent iterations of achievement goal theory have merged the approach‐avoidance perspective with achievement goals, resulting in different types of achievement goals (Cury et al., 2002; Elliot et al., 2011; Elliot & McGregor, 2001). In this study, we focus particularly on mastery‐approach goals, which pertain to developing competence relative to self‐set standards (Elliot & Hulleman, 2017).

Unlike performance goals, which prioritize external comparisons and evaluations (Martin & Elliot, 2016), the pursuit of mastery‐approach goals is facilitated by having a growth mindset. Students with a growth mindset perceive ability as malleable and capable of growth through effort and practice (Weissman & Elliot, 2023; Yeager & Dweck, 2020). Consequently, learners with mastery‐approach goals are persistent, value the learning process and are growth‐oriented (Huang, 2011; Hulleman et al., 2010; Payne et al., 2007). Previous studies have devoted a significant amount of effort to exploring the factors that shape mastery‐approach goals, with a particular emphasis on the importance of the social context (Warburton, 2017). For example, supportive teachers who emphasize learning over performance facilitate the pursuit of mastery‐approach goals (Liem et al., 2016; Matos et al., 2017; Turner et al., 2013).

### Peer cooperation, competition and mastery‐approach goal

Peers exert a powerful influence on students' motivation (Rudolf & Lee, 2023; Wentzel, 2022). Students' relationships with their peers can be characterized as either cooperative (which we refer to as peer cooperation) or competitive (which we refer to as peer competition) (Roseth et al., 2008).

Past studies on peers have traditionally examined peer influences at the individual level (e.g., Rudolf & Lee, 2023; Wentzel et al., 2014). Individual‐level perceptions of peer cooperation or competition refer to students' subjective perceptions of the nature of their peer interactions (Branco, 2003; Fülöp, 2022). Even within the same classroom or school, students can report varying experiences of their peers as the focus is on individuals' subjective perceptions of the environment (James et al., 2008). For example, Student A and B might both be in the same school, but Student A might perceive her peers to be relatively more competitive than Student B depending on her subjective perceptions of her interactions with them. Previous research found that students' perceptions of their peers are associated with their achievement goals (Roussel et al., 2011; Wentzel et al., 2014). For example, Hemi (2021) found that students' achievement goals (e.g., mastery‐approach goals) were significantly predicted by their perception of peers' achievement goals. In another study, Mouratidis et al. (2018) found that students who perceived their teachers and peers to be more focused on learning and personal development were also more likely to pursue mastery‐approach goals.

Despite considerable heterogeneity in students' subjective perceptions, a shared school climate can emerge at the group level (Rudasill et al., 2018; Wang & Degol, 2016). Educational researchers have demonstrated that group‐level (e.g., classroom or school level) characteristics were also significantly associated with individual‐level student outcomes (e.g., Konishi et al., 2017; Marsh et al., 2009; Morin et al., 2014). Hence, whatever consensus can be achieved at the school level can be used to distinguish one school from another (Lüdtke et al., 2009; Marsh et al., 2009). For example, despite variability in individual perceptions of the different students in School A, as a whole, School A might have a more cooperative peer climate compared to School B (e.g., Wang & Dishion, 2012). Existing studies on peers have mostly focused on students' individual perceptions of their peers, but relatively less attention has been devoted to peer cooperation and competition.

Importantly, research has shown that both individual‐level perceptions and aggregated perceptions can play unique roles in students' values and achievement goals (Dicke et al., 2021; Lam et al., 2015). Analytical approaches such as multilevel frameworks (e.g., multilevel structural equation modelling) allow the simultaneous examination of both individual‐level and aggregated school‐level perceptions (Bardach, Oczlon, et al., 2020; Bardach, Yanagida, & Lüftenegger, 2020; Li et al., 2023; Marsh et al., 2009). These findings support the conceptualization of peer cooperation and competition at both the individual and school levels. Hence, we explored both individual‐level and school‐level peer cooperation and competition and how they are associated with students' mastery‐approach goals.

### Peer cooperation and mastery‐approach goals

Peer cooperation is characterized by collaborative and supportive student interactions (Abercrombie et al., 2019; Endedijk et al., 2015). It promotes a shared focus on learning and understanding, fosters intrinsic motivation and enhances academic achievement through positive interpersonal dynamics (Martin & Dowson, 2009). Previous research has shown that in environments where learning and self‐improvement are valued, students are more likely to adopt mastery‐approach goals (King & Mendoza, 2020; Michou et al., 2013; Warburton, 2017). Given that mastery‐approach goals are characterized by a focus on acquiring knowledge and developing skills rather than seeking external validation or engaging in social comparison (Elliot et al., 2011; King et al., 2024), this aligns with the understanding that mastery‐oriented climates, like those fostered by peer cooperation, encourage students to engage deeply with the material, seek challenges and view mistakes as opportunities for growth (Anderman & Anderman, 2009). Hence, we posited the following hypothesis:

Hypothesis 1 (H1) *Individual‐level peer cooperation will be positively associated with mastery‐approach goals*.

The school context exerts a substantial influence on student outcomes by creating a social arena where peer interactions can profoundly impact motivation. Though research on school‐level peer cooperation is relatively less extensive, the significance of aggregated school‐level factors that reflect the school environment has been well‐documented (e.g., Dicke et al., 2021; Konishi et al., 2017; Li et al., 2023). For instance, Dicke et al. (2021) found that school‐level achievement impacts both individual and school‐level self‐concept. Additionally, Konishi et al. (2017) highlighted that school‐level peer support and fairness are significant predictors of bullying. Similarly, Li et al. (2023) demonstrated that both individual and school‐level expectancy and value beliefs are associated with students' reading achievement. These findings underscore the pervasive influence of school‐level factors on various aspects of students' outcomes, suggesting that similar mechanisms may support school‐level peer cooperation. Thus, we posit:

Hypothesis 2 (H2) *School‐level peer cooperation will be positively associated with mastery‐approach goals*.

### Peer competition and mastery‐approach goals

Peer competition is traditionally viewed through the lens of relative performance among peers, where students engage in social comparison, typically aligning with performance‐approach and performance‐avoidance goals (Elliot & Sommet, 2023; Johnson & Johnson, 2009; Murayama & Elliot, 2012). Most previous research linked such competition to deleterious academic and psychological outcomes. For instance, heightened competition has been associated with increased anxiety, elevated stress and burnout among students, as the focus on outperforming others can be debilitating (Skaalvik & Skaalvik, 2017; Van Yperen & Leander, 2014). This emphasis on relative standing also correlates with lower life satisfaction and diminished well‐being (Roseth et al., 2008; Rudolf & Lee, 2023; Wang et al., 2024), as students can experience a constant fear of being outperformed.

Moreover, peer competition has been shown to erode students' academic integrity and sense of belonging. In highly competitive settings, students may resort to cheating and dishonesty to secure an edge over their peers (Anderman & Murdock, 2011; Li et al., 2022; Wenzel & Reinhard, 2020). Such behaviours are fuelled by the perception that academic success hinges predominantly on outperforming others, thus shifting focus away from genuine mastery of the material (Butler, 1992, 1995). Over time, peer competition, reinforced by competitive ranking or public recognition of top performers, can further exacerbate these adverse effects and undermine intrinsic motivation to learn (Bardach, Oczlon, et al., 2020; Bardach, Yanagida, & Lüftenegger, 2020; Grant & Shandell, 2022; Hart & Rodgers, 2024). Consequently, when peers emphasize interpersonal competition, mastery‐approach goals may be compromised, as students become more concerned with surpassing others' performance than with improving their own knowledge and skills (Butler, 1992, 1995; Van Yperen & Leander, 2014).

Nevertheless, some studies point to the possibility that peer competition can yield adaptive outcomes under certain circumstances. For example, Bardach, Oczlon, et al. (2020) and Bardach, Yanagida, and Lüftenegger (2020) found a small yet significant positive correlation between peer competition and mastery‐approach goals, suggesting that when competition is construed as a challenge to enhance personal excellence, it can motivate self‐improvement (Deci & Moller, 2005; Johnson & Johnson, 1989). Qualitative evidence indicates that students who perceive competition as an avenue to improve their abilities may persist through difficulties and cultivate a mastery orientation (Fülöp, 2004, 2022; Fülöp et al., 2025; King et al., 2012). Despite the substantial evidence demonstrating the stress‐inducing and potentially unethical aspects of peer competition, it is plausible that, on balance, peer competition is more likely to hinder mastery‐oriented goals than to facilitate them.

Drawing on these mixed findings, we posit that in most typical school contexts, the negative facets of peer competition are likely to predominate, thereby detracting from the pursuit of mastery‐approach goals at both individual and school levels. A competitive peer context may compel students to prioritize external validation over personal growth, dampening the intrinsic motivation and deep engagement necessary for mastery‐approach goals (Anderman & Murdock, 2011; Murayama & Elliot, 2012; Skaalvik, 2020). Thus, we articulate two additional hypotheses:

Hypothesis 3 (H3) *Individual‐level peer competition will be negatively associated with mastery‐approach goals*.

Hypothesis 4 (H4) *School‐level peer competition will be negatively associated with mastery‐approach goals*.

### Controlling for covariates

To ensure that the results were not due to unmeasured third‐variable confounds, we considered various covariates. Individual‐level covariates, such as gender, age, grade and socioeconomic status (SES), as well as school‐level covariates, such as school type, school size and school SES. These factors have been reported as correlates of mastery‐approach goals (Huang, 2011, 2012). Previous studies have demonstrated that individual motives such as work mastery (intrinsic motivation to exert effort and a preference for seeking out difficult, challenging tasks) and fear of failure (desire to avoid failure and its associated negative consequences) are associated with mastery‐approach goals (Baranik et al., 2010; Guo et al., 2023). Including these motives (work mastery and fear of failure) and demographic factors as covariates helps safeguard against the possibility of uncontrolled third‐variable influences confounding the associations among the key variables.

Peer competition is theoretically assumed to link more with performance‐approach and avoidance goals than with mastery‐approach goals (Elliot & Sommet, 2023; Murayama & Elliot, 2012). This is attributed to the pressures associated with competition as students focus on outperforming others relative to emphasizing authentic learning, which may result in a shared covariance on the consequences of peer cooperation and competition. One key limitation of the PISA 2018 dataset is that it does not include direct measures of performance‐approach goals. However, it does include a variable called competitiveness, which we used as a proxy measure of performance‐approach goals (OECD, 2019).

### The present study

The present study examines the associations among peer cooperation, peer competition and mastery‐approach goals. We tested the following hypotheses using a cross‐cultural dataset drawing on 80 countries^1^:

*Individual‐level peer cooperation will be positively associated with their mastery‐approach goals*.

*School‐level peer cooperation will be positively associated with students' mastery‐approach goals*.

*Individual‐level peer competition will be negatively associated with mastery‐approach goals*.

*School‐level peer competition will be negatively associated with mastery‐approach goals*.

In addition, we incorporated a diverse range of covariates at both the individual (e.g., fear of failure, work mastery and demographic factors) and school (e.g., school type, school size and school socioeconomic status) levels and we utilized a proxy measure of performance‐approach goals as an external covariate outcome to assess the robustness of our findings. We also conducted supplementary analyses to explore the extent to which the results generalized across diverse cultural contexts.

## METHOD

### Data and measures

The present study employed data from the Programme for International Student Assessment (PISA) 2018,^2^ a large‐scale international assessment conducted by the Organization for Economic Co‐operation and Development (OECD). Originally, the PISA 2018 dataset encompassed responses from 612,004 adolescents across 80 countries. However, certain countries^3^ were excluded due to the lack of data on critical variables in their datasets. Post these exclusions, this study included responses from 565,732 15‐year‐old adolescent students nested within 20,227 schools across 75 countries. The average number of students per school was 27.97. The gender ratio was nearly equal: males (281,552; 49.8%) and females (281,552; 50.2%).

#### Mastery‐approach goals

Mastery‐approach goals were measured using the mastery‐approach scale in PISA 2018. It contains three items and is rated on a 4‐point scale from 1 (*not at all true for me*) to 4 (*extremely true for me*). This scale was adapted and developed based on the Achievement Goal Questionnaire Revised (Elliot & Murayama, 2008). A sample item is, “My goal is to learn as much as possible.”

#### Individual‐level peer cooperation and competition

This study used two distinct four‐item measures of individual‐level cooperation (e.g., ‘[In my school] it seems that students are cooperating with each other’) and competition (e.g., ‘[In my school] it seems that students are competing with each other’); response options ranged from 1 = *not at all true* to 4 = *extremely true*. The PISA team adapted these measures from the Work and Family Orientation Scale (Helmreich et al., 1978) and assessed the degree to which students at school compete with or cooperate with each other (OECD, 2019).

#### School‐level peer cooperation and competition

We used the latent aggregation of students' peer cooperation and competition to operationalize school‐level peer cooperation and competition, respectively. Doing so follows prior empirical precedent in PISA, where individual‐ and school‐level variables are used (Göllner et al., 2018; Marsh et al., 2009). As in traditional CFA models, the scores on items were utilized to form latent factors and peer cooperation and competition scores by students within the same school were employed to form latent school‐level peer cooperation and competition.

#### Covariates

##### Fear of failure

Fear of failure was measured using the index of fear of failure provided in the PISA 2018 dataset. This index contains three items and measures students' fear of failure using a 4‐point scale from 1 (*strongly disagree*) to 4 (*strongly agree*). The OECD (2019) adapted and developed this scale based on the Performance Failure Appraisal Inventory (Conroy et al., 2002). A sample item is, “When I am failing, this makes me doubt my plans for the future.”

##### Work mastery

Work mastery was measured using the index of work mastery provided in the PISA 2018 dataset. This index contains four items and measures students' achievement motivation, and is measured using a 4‐point scale from 1 (*strongly disagree*) to 4 (*strongly agree*). This scale was adapted and developed partly based on the Work and Family Orientation Scale (Helmreich et al., 1978). A sample item is, “I find satisfaction in working as hard as I can”.

##### Proxy measure of performance‐approach goals

We employed the measures of competitiveness in the PISA 2018 dataset (OECD, 2019) as a proxy measure of performance‐approach goals,^4^ and it is measured using a 4‐point scale from 1 (*not at all*) to 4 (*extremely true*). The items are “I enjoy working in situations involving competition with others”, “It is important for me to perform better than other people on a task” and “I try harder when I'm in competition with other people.”

##### Demographic factors

At the student level, gender (*female* = 0 and *male* = 1), grade level, and SES were included as covariates. PISA defines “economic and social‐cultural status” as a measure of SES, encompassing data on students' family backgrounds, such as the number of books at home and their parents' education and occupation (OECD, 2019). At the school level, school type (0 = *private school*, 1 = *public school*), school size, and school SES were included as covariates. The school SES was derived from the aggregated student‐level SES within each school.

### Data analysis

#### Preliminary analysis

The proportions of missing data for all items ranged from 0.0% for gender to 26.8% for the item “Students feel that they are encouraged to cooperate with others”. The study used Markov Chain Monte Carlo (MCMC) imputation to deal with the missing data (Soley‐Bori, 2013). MCMC has a high accuracy rate in imputing missing data, which enables us to analyse complex data (Marjoram et al., 2003). All items were standardized to a mean of zero and a unit SD to facilitate the interpretation of results and reduce nonessential multicollinearity (Marjoram et al., 2003).

We conducted a series of multilevel confirmatory factor analyses (ML‐CFAs) to validate the measurement models of the focal variables and to examine the bivariate correlations among them. Initially, an ML‐CFA was performed specifically for mastery‐approach goals to ensure the construct's validity at both the student and school levels. Subsequently, another ML‐CFA was employed to test the bivariate correlations among the substantive factors and covariates of the hypothesized model.

Intraclass correlation coefficients (ICC), specifically ICC1 and ICC2 for mastery‐approach goals, peer cooperation and peer competition, were computed to detect the appropriateness of doubly latent ML‐SEM. ICC1 quantifies the extent to which variance in student‐level data is attributable to school‐level differences, while ICC2 assesses the reliability of aggregated student‐level data (Lüdtke et al., 2008). Previous studies have suggested that ICC1 values greater than .05 and ICC2 values higher than .60 justify the use of multilevel modelling (Gonzàlez‐Romà & Hernàndez, 2017; Lüdtke et al., 2008, 2011).

#### Primary analysis

The primary analysis involved the doubly latent ML‐SEM approach^5^, which accounts for individual and school‐level factors, providing a robust framework for understanding these dynamics (Marsh et al., 2009; Morin et al., 2014). This approach controls for measurement error at both levels and corrects for sampling error in the aggregation process, ensuring more accurate estimates of the contextual‐level effects. Although our focal variables were specified at the individual and school levels, level 3 (country level) was included to account for the clustering of students within schools, and schools within countries. Doing so aligns with the international sampling design of PISA.

The doubly latent ML‐SEM involves four steps, progressing from basic to more sophisticated models. The first step utilized an initial multilevel structural equation model (SEM), aggregating peer cooperation and competition at the individual level as latent factors. In the second step, we employed the basic model of the doubly latent SEM, which involved the latent school‐level factors of peer cooperation and competition by aggregating student scores within each school. The third step integrated additional multilevel covariates (e.g., motives and demographic factors) into the previous basic model to enhance the robustness of the analysis. In the final step, we added a proxy measure of performance‐approach goals as an additional outcome to have a more nuanced understanding of how peer cooperation and competition might be associated with mastery‐approach goals when the proxy measure of performance‐approach goals was involved.

The final student weight was employed to adjust for the unequal chances in sampling selection. Root Mean Square Error of Approximation (RMSEA), Standardized Root Mean Residual (SRMR), Comparative Fit Index (CFI) and Tucker‐Lewis Index (TLI) were used to evaluate the model fit, with TLI and CFI above .90, and RMSEA and SRMR below .10 were considered acceptable fits (Hu & Bentler, 1999).

In terms of sample size, it is recommended that there be at least 50 but ideally 100 level 2 (i.e., school level) units. Furthermore, it is recommended that there be at least 10–15 students per unit so as to avoid nonconvergence and estimation errors (Lüdtke et al., 2008, 2011). The present study meets these requirements, encompassing more than 20,000 schools, with each school having approximately 28 students, nested within 75 countries. This study employed the following benchmarks to interpret effect sizes: *β* values of .05, .10 and .25 were classified as small, medium and large effects (Keith, 2019).

## RESULTS

### Preliminary analyses

#### Descriptive analysis

The descriptive analysis of substantive factors and related items is shown in Table 1.

**TABLE 1:** Descriptive statistics for the focal variables.

Factors	Items	*α*	Mean	SD	Skewness	Kurtosis
Mastery‐approach goals		.86	3.49	.99		
	ST208Q01HA		3.48	1.13	−.35	−.63
	ST208Q02HA		3.35	1.13	−.24	−.69
	ST208Q04HA		3.63	1.11	−.52	−.45
Peer cooperation		.91	2.69	.73		
	ST206Q01HA		2.63	.84	−.13	−.55
	ST206Q02HA		2.71	.79	−.19	−.40
	ST206Q03HA		2.69	.82	−.19	−.46
	ST206Q04HA		2.72	.84	−.22	−.51
Peer competition		.84	2.56	.72		
	ST205Q01HA		2.53	.84	.04	−.60
	ST205Q02HA		2.58	.86	−.03	−.67
	ST205Q03HA		2.47	.87	.02	−.69
	ST205Q04HA		2.64	.93	−.10	−.86
Fear of failure		.80	2.56	.79		
	ST183Q01HA		2.57	.94	−.19	−.85
	ST183Q02HA		2.56	.91	−.13	−.78
	ST183Q03HA		2.54	.96	−.09	−.95
Work mastery		.78	3.03	.60		
	ST182Q03HA		3.06	.79	−.72	.32
	ST182Q04HA		2.96	.77	−.43	−.11
	ST182Q05HA		3.18	.73	−.79	.78
	ST182Q06HA		2.90	.81	−.44	−.22

#### ML‐CFAs

The initial ML‐CFA conducted for mastery‐approach goals demonstrated a good model fit: *χ* ^2^ (3) = 8491.65, CFI = .97; TLI = .94; RMSEA = .07; SRMR L1 = .01, SRMR L2 = .29. Next, the ML‐CFA involving all substantive factors demonstrated an excellent fit: *χ* ^2^ (171) = 83690.98, CFI = .97; TLI = .963; RMSEA = .03; SRMR L1 = .02, SRMR L2 = .06. Correlations of all the tested variables are reported in Table 2. Peer cooperation (*β* = .27, *p* < .001 and *β* = .47, *p* < .001) and peer competition (*β* = .19, *p* < .001 and *β* = .49, *p* < .001) at both individual and school levels were positively correlated with mastery‐approach goals.

**TABLE 2:** Bivariate correlations among the variables.

		1	2	3	4	5	6	7	8	9	10
1	Mastery‐approach goals	1	.27***	.19***	.07***	.46***	−.09***	−.01***	.09***	–	–
2	Peer cooperation	.47***	1	.23***	−.01***	.27***	.01***	.03***	.05***		
3	Peer competition	.49***	.47***	1	.11***	.19***	.06***	−.01***	.08***	–	–
4	Fear of failure	–	–	–	1	.09***	−.14***	−.00*	−.01***	–	–
5	Work mastery	–	–	–	–	1	−.07***	.00***	.05***	–	–
6	Gender	–	–	–	–	–	1	.01	.01***	–	–
7	Grade	–	–	–	–	–	–	1	.03***	–	–
8	ESCS	−.28***	.11***	−.08***	–	–	–	–	1	–	–
9	School type	−.01	−.06***	−.08***	–	–	–	–	−.29***	1	–
10	School size	.05	.11***	.16***	–	–	–	–	.11***	−.07***	1

*Note*: Above are the correlations among variables at the student level; below are the correlations among variables at the school level.

Abbreviation: ESCS, socioeconomic status.

*p < .05; **p < .01; ***p < .001.

#### ICCs

For mastery‐approach goals, ICC1 was .11, indicating that 11% of the variance was due to differences between schools, while an ICC2 of .82 reflected a high level of group‐level consistency. Similarly, for peer cooperation, ICC1 was .07 and ICC2 was .69, and for peer competition, ICC1 was .07 and ICC2 was .68, also indicating meaningful between‐school differences and reliable group‐level data. These results justify sufficient between‐level variance in the aggregated data and ensure the appropriateness of using multilevel modelling (Morin et al., 2014).

### Primary analyses

#### Doubly latent ML‐SEM

##### Step 1: Individual‐level peer cooperation and competition

The initial model fit well: *χ* ^2^ (97) = 8405.70, CFI = .93; TLI = .92; RMSEA = .01; SRMR for level 1 (L1) = .03, SRMR for level 2 (L2) = .65, SRMR for level 3 (L3) = .49. Mastery‐approach goals were positively associated with individual‐level peer cooperation (*β* = .24, *p* < .001, medium effect) and competition (*β* = .16, *p* < .001, medium effect).

##### Step 2: School‐level peer cooperation and competition

The basic model which only included the focal variables showed a satisfactory model fit: *χ* ^2^ (86) = 7434.13, CFI = .94; TLI = .93; RMSEA = .01; SRMR for L1 = .03, SRMR L2 = .08, SRMR L3 = .49. The results showed that mastery‐approach goals were positively associated with individual‐level (*β* = .21, *p* < .001, medium effect) and school‐level peer cooperation (*β* = .44, *p* < .001, large effect), supporting our H1 and H2. Individual‐level peer competition (*β* = .13, *p* < .001, medium effect) and school‐level peer competition were also positively associated with mastery‐approach goals (*β* = .17, *p* < .001, medium effect), rejecting our H3 and H4.

##### Step 3: Full model with covariates

**FIGURE 1:**
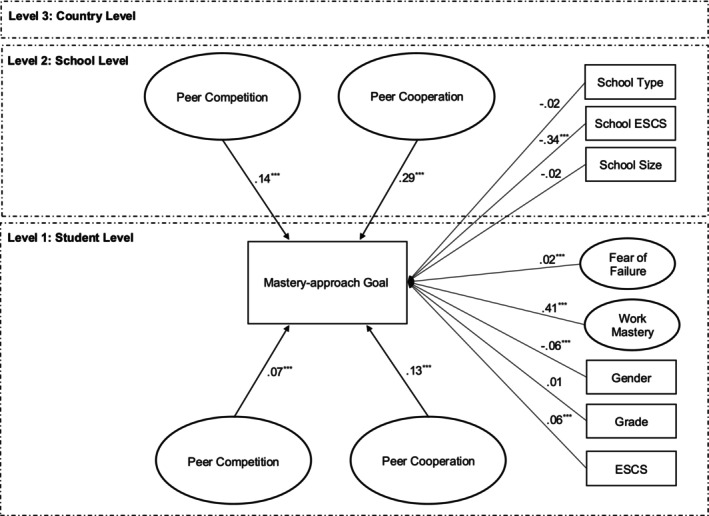
Doubly latent multilevel structural equation modelling with covariates. ***p < .001; ESCS, student socioeconomic status; School ESCS, school socioeconomic status.

The full model demonstrated a good model fit: *χ* ^2^ (225) = 14,958.01, CFI = .95; TLI = .94; RMSEA = .01; SRMR L1 = .03, SRMR L2 = .09, SRMR L3 = .46. As shown in Figure 1, at the student level, both individual‐level peer cooperation (*β* = .13, *p* < .001, medium effect) and peer competition (*β* = .07, *p* < .001, small effect) were positively associated with mastery‐approach goals. At the school level, mastery‐approach goals were also significantly associated with school‐level peer cooperation (*β* = .29, *p* < .001, large effect) and competition (*β* = .14, *p* < .001, medium effect).

##### Step 4: Including competitiveness as a proxy for performance‐approach goals

**FIGURE 2:**
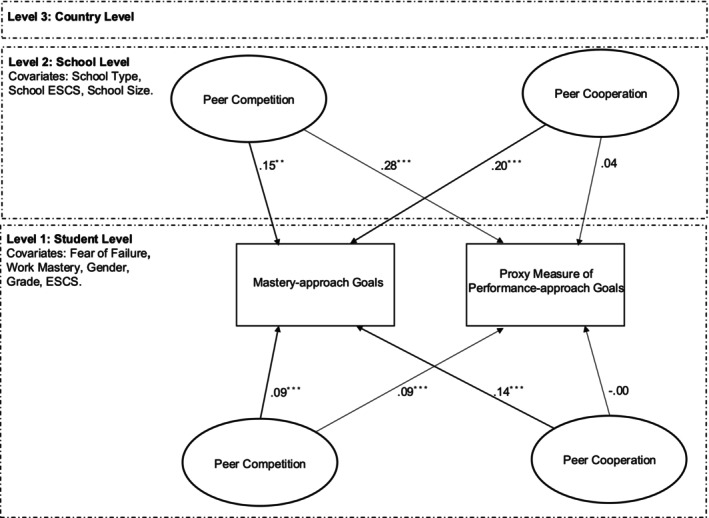
Doubly latent multilevel structural equation modelling of peer cooperation and competition on master‐approach goals and the proxy measure of performance‐approach goals. **p < .01; ***p < .001; ESCS, student socioeconomic status; School ESCS, school socioeconomic status.

As shown in Figure 2, the full model with proxy for performance‐approach goals demonstrated satisfying model fit: *χ* ^2^ (307) = 303,141.68, CFI = .95; TLI = .94; RMSEA = .01; SRMR L1 = .03, SRMR L2 = .09, SRMR L3 = .45. As shown in Figure 2, both individual‐level peer cooperation (*β* = .14, *p* < .001, medium effect) and competition (*β* = .09, *p* < .001, small effect) were positively associated with mastery‐approach goals. Meanwhile, the proxy for performance‐approach goals was only positively associated with individual‐level peer competition (*β* = .09, *p* < .001, small effect), but not with individual‐level peer cooperation.

At the school level, mastery‐approach goals were also significantly associated with school‐level peer cooperation (*β* = .20, *p* < .001, medium effect) and competition (*β* = .15, *p* < .001, medium effect). Additionally, only school‐level peer competition was significantly associated with the proxy for performance‐approach goals (*β* = .28, *p* < .001, large effect).

### Supplementary analyses

We conducted three additional analyses to complement the primary results. First, we conducted supplementary analyses to examine the associations among peer cooperation, competition, and mastery‐approach goals across 75 countries to explore whether the results were generalizable across contexts (Tables S1–S3).

Second, considering between‐country differences, we explored variations in the magnitude of associations among peer cooperation, competition, and mastery‐approach goals at both the individual and school levels. To examine this, we employed multilevel structural equation modelling with random slopes, allowing for individual and school‐level peer cooperation and competition to vary at the country levels (Figure S1).

Third, to elucidate potential cultural similarities and differences, we compared collectivist and individualist contexts to determine whether the associations remain consistent across different cultural settings. Specifically, we used the multilevel multigroup doubly latent ML‐SEM in collectivist (Figure S2) and individualist countries (Figure S3) for a rigorous examination of whether the hypothesized associations hold across cultural contexts.^6^

## DISCUSSION

The present study examines the associations among peer cooperation, peer competition, and mastery‐approach goals. Supporting H1 and H2, the results revealed that both individual and school‐level peer cooperation were positively associated with mastery‐approach goals.

The findings on peer competition did not align with our expectations. Both individual and school‐level peer competition were positively associated with students' mastery‐approach goals, failing to support H3 and H4. The results were robust even after controlling for individual motives and demographic factors as covariates, as well as a proxy measure of performance‐approach goals.

### Peer cooperation and mastery‐approach goals

The present study contributes to achievement goal theory by confirming the positive association between individual‐level peer cooperation and mastery‐approach goals with a medium effect size. This finding aligns well with the existing literature, which reports medium to large effect sizes for cooperation on various positive educational outcomes, including academic engagement and well‐being (King et al., 2024; Rudolf & Lee, 2023; Van Ryzin & Roseth, 2021; Wang et al.,^2024^). Our findings reinforce the robust positive association between peer cooperation and mastery‐approach goals.

School‐level peer cooperation was also positively associated with mastery‐approach goals, with a large effect size. This underscores the necessity of accounting for school‐level contexts in studies of peer climate (Marsh et al., 2009; Rudasill et al., 2018). When comparing the multilevel associations between peer cooperation and mastery‐approach goals, the findings suggested that mastery‐approach goals are positively associated with both individual and school‐level peer cooperation. Peer climates represent a crucial socio‐motivational component (Skinner et al., 2022; Wentzel & Skinner, 2022). Hence, focusing on peers seems to be an important extension of the existing literature, which has mostly focused on the role of internal psychological factors and the role of teachers.

### Counterintuitive findings on peer competition

Surprisingly, we found that both individual‐ and school‐level peer competition were positively associated with mastery‐approach goals, albeit with medium effect sizes. The extant literature generally holds a predominantly negative view of competition, often conceptualizing it as a zero‐sum game that undermines intrinsic motivation and fosters a win‐lose mentality (Butler & Kedar, 1990; Chun et al., 2019; Lam et al., 2004). While the traditional conceptualization of competition emphasizes its maladaptive aspects, there is growing evidence that it can also have positive effects, such as stimulating higher levels of effort, creativity and persistence (Elliot, 2020; Fülöp, 2022; Fülöp et al., 2025; Murayama et al., 2021; Watkins, 2007).

For instance, Johnson and Johnson (1989) argued that competitive environments could stimulate higher levels of effort, creativity and persistence, which are essential for mastering new skills and knowledge. Supporting this view, a qualitative study found that many students (69%) viewed improvement as the most important function of competition (Fülöp, 2004). Fülöp (2004) suggested that competition can accelerate the development of personal abilities, as some students view it as a means of realizing their full potential and enhancing competencies. Deci and Moller (2005) further explained that perceiving competition as energizing can boost intrinsic motivation, as it underscores individuals' efforts to see themselves as agents of personal growth. Empirical studies have also confirmed that competitive environments can enhance task enjoyment and intrinsic motivation (Song et al., 2013; Tauer & Harackiewicz, 2004).

Although most studies suggest that peer competition is not theoretically associated with mastery‐approach goals, a handful of studies have reported positive associations between competition‐related factors and mastery‐approach goals. For example, King et al. (2012) found that having a competitive attitude can positively drive the adoption of mastery‐approach goals, particularly in contexts where students view competition as a means to grow and improve alongside their peers. This also aligns with Guo et al.'s (2023) finding of a small positive association between competitiveness and mastery‐approach goals (*β* = .08) in a cross‐cultural study.

Our findings about the positive associations between peer competition and mastery‐approach goals highlight the need to consider the context and framing of competition in educational settings and caution against overly simplistic views of competition (see also Elliot, 2020; Murayama et al., 2021). Perceived environmental competition inherently possesses neither positive nor negative qualities regarding psychological functioning; instead, its effects are contingent upon the objectives pursued in response to competition‐driven concerns (Elliot, 2020; Young & Elliot, 2020).

Nevertheless, a more cautious interpretation of these findings is needed. While peer competition was positively associated with mastery‐approach goals, it may also promote performance goals (e.g., Murayama & Elliot, 2012). Peer competition might also potentially lead to maladaptive outcomes such as dishonesty, obstructing others, or withholding help (Daumiller & Janke, 2019; David et al., 2021; Roseth et al., 2008). This raises concerns about the competition's overall value, as it may come at the expense of cooperation and mutual support (Butler, 1992). Therefore, although our findings suggest that peer competition is positively associated with mastery‐approach goals, it is important to acknowledge that peer competition may have both positive and negative effects, depending on how it is framed and perceived by students. More research is needed that considers multiple types of achievement goals to have a more comprehensive understanding of the role of peer competition.

### Theoretical and practical implications

This study contributes to the existing literature with theoretical and practical implications. Theoretically, the current study contributes to the existing literature on mastery‐approach goals by highlighting the important role played by peers. Whereas previous research has predominantly concentrated on the roles of teachers and internal psychological factors, this investigation reveals the important roles played by peer cooperation and competition in relation to mastery‐approach goals. These peer‐related factors are significant and comparable to the influences arising from teacher‐related factors (e.g., teacher autonomy‐support, *β* = .19; Benita & Matos, 2021) and other internal psychological factors (e.g., trait conscientiousness, *r* = .32; Payne et al., 2007), underscoring the considerable role that peers play alongside other potential antecedents of achievement goals. Moreover, the persistence of robust effects, even after adjusting for covariates, indicates that peers distinctly contribute to the pursuit of mastery‐approach goals, independent of other critical covariates, including work mastery, fear of failure and demographic variables.

Practically, this study suggests the importance of peers in achievement goal pursuit, potentially guiding their academic development towards mastery. The current findings revealed a coexistence of cooperation and competition, rather than viewing them as mutually exclusive (Charleton et al., 2018; Chen et al., 2011). This is crucial for understanding how peer competition and cooperation can function as complementary rather than conflicting forces within educational settings. Educators should pay close attention to fostering a balanced climate of peer cooperation and competition, as both are crucial in steering students towards adopting mastery‐approach goals.

### Limitations and future directions

Despite the aforementioned implications, it is important to acknowledge certain limitations within this study. Firstly, the cross‐sectional nature of the PISA data precludes the establishment of causality. While large‐scale datasets such as PISA offer the advantage of broad generalizability due to their extensive sample sizes across 75 countries, they also come with methodological constraints. Specifically, the cross‐sectional nature of the data limits the ability to establish temporal order, which is necessary for causal claims (see Guo et al., 2023, for a similar discussion). To strengthen the validity of current findings, future research should incorporate longitudinal or experimental designs to better examine causal relationships.

Secondly, this study exclusively examines the direct associations among peer cooperation, competition and mastery‐approach goals without considering potential mediating or moderating variables within those associations. As an initial foray into understanding the associations among peer cooperation, peer competition and mastery‐approach goals, future research is urged to investigate a broader range of factors that influence students' mastery‐approach goals. This would help reveal the complex dynamics through which peer climates shape students' orientations towards mastery goals.

Third, this study exclusively employed a quantitative methodology. Future research could benefit from integrating qualitative approaches, such as interviews. This would provide more nuanced insights into how peers actually perceive competition. It is possible that some students might have more positive views of peer competition than others (Fülöp, 2004). Doing so would yield a deeper understanding of the contextual‐level factors and personal experiences shaping achievement goals.

Fourth, in this study, we only focused on individual‐level and school‐level peer cooperation and competition. This is an inherent limitation, as there might be significant variability within classrooms where peer interactions might be more frequent. However, as PISA does not contain classroom‐level data, we were unable to test this hypothesis. We suggest future studies include individual, class, and school‐level data on peer cooperation and competition to develop a more nuanced understanding of how these dynamics operate across different levels of analysis.

Fifth, although PISA employs a stratified two‐stage sampling design to enhance representativeness (OECD, 2019), some schools in the 2018 dataset include only a small number of students. This limited within‐school sample size may reduce the reliability and generalizability of aggregated school‐level constructs. Future research could address this issue by recruiting larger within‐school samples or exploring alternative methods of aggregating peer climate data, thereby improving the robustness of school‐level measures.

Sixth, this study primarily focuses on mastery‐approach goals. Other achievement goals, such as mastery‐avoidance, performance‐approach and performance‐avoidance goals, were not directly examined. Although we included supplementary analyses using a proxy measure of performance‐approach goals, PISA does not have an exact measure of performance‐approach. Future research can include direct assessments of different types of achievement goals.

Last, our cross‐cultural analysis in supplementary analyses was limited to a median split based solely on Hoftede et al.'s (2010) individualism–collectivism dimension, which may oversimplify cultural influences. Achievement motivation varies across multiple cultural dimensions (e.g., power distance and uncertainty avoidance) and median splits might obscure meaningful cultural variability. Future studies should adopt more comprehensive theoretical frameworks and rigorous methodologies to capture nuanced cross‐cultural differences in how peer cooperation and competition relate to achievement goals.

## CONCLUSION

This study underscores the significance of peers in understanding achievement goal pursuit. Drawing on a cross‐cultural dataset covering 75 countries across the world, we found that both individual‐ and school‐level peer cooperation were positively associated with mastery‐approach goals pursuit. Furthermore, we also found that individual and school peer competition was also positively associated with mastery‐approach goals, albeit the associations were weaker than those compared to peer cooperation. While caution is warranted when interpreting the results of peer competition, dismissing it entirely as negative might be somewhat simplistic. Overall, this study emphasizes the importance of expanding our theoretical understanding to account for the role of peers in students' achievement goal pursuit.

## AUTHOR CONTRIBUTIONS

**Yikang Chen:** Methodology; writing – original draft; writing – review and editing; conceptualization; validation; investigation; formal analysis; data curation. **Jiajing Li:** Writing – review and editing; methodology; validation. **Harold Chui:** Supervision; writing – review and editing. **Ronnel B. King:** Conceptualization; methodology; supervision; writing – review and editing; writing – original draft; investigation.

## CONFLICT OF INTEREST STATEMENT

The authors have no conflicts of interest to declare.

## Supporting information


Data S1:

## Data Availability

The data that support the findings of this study are openly available in PISA 2018 Database at https://www.oecd.org/pisa/data/2018database/.

## Footnotes

^1^
We use the term “country” for shorthand, but note that some of the contexts included in the PISA dataset are more appropriately classified as cities, jurisdictions or regions (e.g., Hong Kong Special Administrative Region of the People's Republic of China).

^2^
PISA 2022 is the latest round of PISA, but it does not include items specifically measuring mastery‐approach goals. Hence, we used PISA 2018.

^3^
Excluded countries were Canada, Israel, Lebanon, Norway, and North Macedonia.

^4^
It is important to note that competitiveness is defined as a disposition that serves an energizing function, and is somewhat distinct from achievement goals, which serve a directional function (Elliot, 2005). According to the hierarchical model of achievement motivation, motives, and goals are conceptually and empirically distinct (Elliot & Sommet, 2023). Although they share conceptual similarities as both emphasize social comparison and performance relative to others, competitiveness is not the same as performance‐approach goals. Thus, we used the term proxy measure of performance goals in the current study to delineate this nuance.

^5^
PISA has a hierarchical structure. Students are nested within schools, and schools are nested within countries. To appropriately address this nested data structure and reduce estimation bias, we adopted a three‐level linear modelling approach. This method enabled us to partition the residual variance at the student (Level 1), school (Level 2), and country (Level 3) levels, which helps mitigate estimation biases associated with nested data structures. While we included country‐level effects in our Level 3 modelling to account for between‐country variability, we did not incorporate specific country‐level variables into our analysis. This decision was made because our research questions focused specifically on variables at the student and school levels.

^6^
We thank the editor and an anonymous reviewer for suggesting this analysis.
