# Supplementary material for: Peer cooperation and competition are both positively linked with mastery‐approach goals: An achievement goal perspective
Source: Br J Educ Psychol. 2025 Jun 9;95(4):941–61. doi: 10.1111/bjep.12784 (PMC12590937; doi:10.1111/bjep.12784)
Supplement: Supplementary file 1 — Data S1: [file BJEP-95-941-s001.docx]

**Online supplementary materials for:**

**Peer cooperation and competition are both positively linked with mastery-approach goals: An achievement goal perspective**

**Part 1: Supplementary Tables**

Table S1. Descriptive Statistics across Countries.

Table S2. Correlations among the Focal Variables across Countries.

Table S3. Multilevel Structural Equation Modeling of the Effects of Peer Cooperation and Competition on Mastery-approach Goals across Countries.

Table S4. Individualist and Collectivist Countries.

**Part 2: Supplementary Figures**

Figure S1. Multilevel Structural Equation Modeling of Peer Cooperation and Competition on Mastery-approach Goals with Random Slopes.

Figure S2. Doubly-latent Multilevel Structural Equation Modeling among the Collectivist Countries.

Figure S3. Doubly-latent Multilevel Structural Equation Modeling among the Individualist Countries

**Part 1: Supplementary Tables**

**Table S1**

*Descriptive Statistics across Countries*

|  |  | Countries | Mean | *SD* | *N* |
| --- | --- | --- | --- | --- | --- |
| Mastery-approach Goals | | 8Albania | 4.03 | .86 | 6051 |
|  |  | 31Baku (Azerbaijan) | 3.85 | 1.00 | 4972 |
|  |  | 32Argentina | 3.18 | 1.07 | 10315 |
|  |  | 36Australia | 3.46 | .91 | 11913 |
|  |  | 40Austria | 3.41 | .94 | 6337 |
|  |  | 56Belgium | 3.49 | .85 | 7596 |
|  |  | 70Bosnia and Herzegovina | 3.58 | 1.05 | 5885 |
|  |  | 76Brazil | 3.91 | .92 | 8521 |
|  |  | 96Brunei Darussalam | 3.51 | .91 | 6260 |
|  |  | 100Bulgaria | 3.15 | 1.09 | 4353 |
|  |  | 112Belarus | 3.28 | .90 | 5642 |
|  |  | 152Chile | 3.72 | .95 | 6528 |
|  |  | 158Chinese Taipei | 3.12 | .98 | 7117 |
|  |  | 170Colombia | 3.85 | .86 | 6568 |
|  |  | 188Costa Rica | 3.90 | .96 | 6405 |
|  |  | 191Croatia | 3.29 | .99 | 6291 |
|  |  | 203Czech Republic | 3.34 | .86 | 6525 |
|  |  | 208Denmark | 3.83 | .82 | 6494 |
|  |  | 214Dominican Republic | 3.85 | 1.01 | 3117 |
|  |  | 233Estonia | 3.21 | .91 | 5077 |
|  |  | 246Finland | 3.33 | .89 | 5265 |
|  |  | 250France | 3.25 | .95 | 5709 |
|  |  | 268Georgia | 3.79 | 1.03 | 4647 |
|  |  | 276Germany | 3.39 | .86 | 4055 |
|  |  | 300Greece | 3.37 | .93 | 6019 |
|  |  | 344Hong Kong | 3.34 | .87 | 5689 |
|  |  | 348Hungary | 3.13 | .94 | 4901 |
|  |  | 352Iceland | 3.61 | .96 | 2999 |
|  |  | 360Indonesia | 3.81 | .82 | 11718 |
|  |  | 372Ireland | 3.30 | .93 | 5366 |
|  |  | 380Italy | 3.20 | .94 | 10629 |
|  |  | 383Kosovo | 3.92 | .92 | 4569 |
|  |  | 392Japan | 3.08 | .94 | 5996 |
|  |  | 398Kazakhstan | 3.83 | .93 | 18183 |
|  |  | 400Jordan | 3.87 | 1.00 | 8490 |
|  |  | 410Korea | 3.41 | 1.01 | 6580 |
|  |  | 428Latvia | 3.07 | .88 | 4967 |
|  |  | 440Lithuania | 3.40 | .97 | 6383 |
|  |  | 442Luxembourg | 3.36 | .96 | 4827 |
|  |  | 446Macao | 3.18 | .89 | 3758 |
|  |  | 458Malaysia | 3.75 | .89 | 5988 |
|  |  | 470Malta | 3.60 | .98 | 3069 |
|  |  | 484Mexico | 3.94 | .81 | 5573 |
|  |  | 498Moldova | 3.48 | .87 | 5188 |
|  |  | 499Montenegro | 3.70 | .99 | 6039 |
|  |  | 504Morocco | 3.66 | 1.01 | 4512 |
|  |  | 528Netherlands | 3.17 | .87 | 3752 |
|  |  | 554New Zealand | 3.48 | .87 | 5864 |
|  |  | 591Panama | 3.95 | 1.03 | 3721 |
|  |  | 604Peru | 3.76 | .89 | 4386 |
|  |  | 608Philippines | 3.73 | .97 | 6939 |
|  |  | 616Poland | 3.40 | .87 | 5450 |
|  |  | 620Portugal | 3.42 | .92 | 5437 |
|  |  | 634Qatar | 3.69 | 1.04 | 11937 |
|  |  | 642Romania | 3.47 | .96 | 4910 |
|  |  | 643Russian Federation | 3.17 | 1.01 | 6992 |
|  |  | 682Saudi Arabia | 3.80 | 1.00 | 5647 |
|  |  | 688Serbia | 3.37 | 1.07 | 5582 |
|  |  | 702Singapore | 3.71 | .79 | 6576 |
|  |  | 703Slovak Republic | 3.06 | .96 | 5429 |
|  |  | 704Vietnam | 2.40 | .82 | 5362 |
|  |  | 705Slovenia | 3.13 | .97 | 5944 |
|  |  | 724Spain | 3.31 | 1.02 | 32988 |
|  |  | 752Sweden | 3.41 | .97 | 5025 |
|  |  | 756Switzerland | 3.39 | .87 | 5086 |
|  |  | 764Thailand | 3.65 | .84 | 8434 |
|  |  | 784United Arab Emirates | 3.74 | 1.02 | 17508 |
|  |  | 792Turkey | 3.32 | 1.06 | 6703 |
|  |  | 804Ukraine | 3.02 | 1.00 | 5794 |
|  |  | 826United Kingdom | 3.32 | .95 | 12729 |
|  |  | 840United States | 3.69 | .94 | 4608 |
|  |  | 858Uruguay | 3.57 | 1.00 | 4104 |
|  |  | 975B-S-J-Z (China) | 3.39 | .85 | 11935 |
|  |  | 982Moscow Region (RUS) | 3.12 | 1.03 | 1886 |
|  |  | 983Tatarstan (RUS) | 3.27 | 1.01 | 5382 |
| Peer Competition |  | 8Albania | 2.92 | .67 | 5910 |
|  |  | 31Baku (Azerbaijan) | 2.70 | .76 | 3743 |
|  |  | 32Argentina | 2.25 | .68 | 8139 |
|  |  | 36Australia | 2.78 | .68 | 10722 |
|  |  | 40Austria | 2.47 | .67 | 5365 |
|  |  | 56Belgium | 2.35 | .70 | 7267 |
|  |  | 70Bosnia and Herzegovina | 2.69 | .71 | 5385 |
|  |  | 76Brazil | 2.56 | .75 | 6679 |
|  |  | 96Brunei Darussalam | 2.75 | .64 | 5017 |
|  |  | 100Bulgaria | 2.50 | .75 | 3693 |
|  |  | 112Belarus | 2.28 | .64 | 5398 |
|  |  | 152Chile | 2.50 | .73 | 5183 |
|  |  | 158Chinese Taipei | 2.72 | .76 | 7045 |
|  |  | 170Colombia | 2.55 | .62 | 5405 |
|  |  | 188Costa Rica | 2.49 | .72 | 6227 |
|  |  | 191Croatia | 2.30 | .76 | 5528 |
|  |  | 203Czech Republic | 2.18 | .64 | 6256 |
|  |  | 208Denmark | 2.38 | .65 | 6177 |
|  |  | 214Dominican Republic | 2.59 | .75 | 1622 |
|  |  | 233Estonia | 2.32 | .62 | 4799 |
|  |  | 246Finland | 2.56 | .61 | 5029 |
|  |  | 250France | 2.42 | .79 | 4802 |
|  |  | 268Georgia | 2.43 | .74 | 4282 |
|  |  | 276Germany | 2.26 | .66 | 2547 |
|  |  | 300Greece | 2.51 | .69 | 5542 |
|  |  | 344Hong Kong | 2.81 | .71 | 5578 |
|  |  | 348Hungary | 2.36 | .71 | 4333 |
|  |  | 352Iceland | 2.57 | .71 | 2451 |
|  |  | 360Indonesia | 2.55 | .66 | 11526 |
|  |  | 372Ireland | 2.69 | .70 | 4663 |
|  |  | 380Italy | 2.35 | .69 | 8888 |
|  |  | 383Kosovo | 2.71 | .69 | 4272 |
|  |  | 392Japan | 2.21 | .73 | 5904 |
|  |  | 398Kazakhstan | 2.52 | .77 | 15912 |
|  |  | 400Jordan | 2.75 | .74 | 7999 |
|  |  | 410Korea | 2.84 | .82 | 6561 |
|  |  | 428Latvia | 2.46 | .64 | 4584 |
|  |  | 440Lithuania | 2.58 | .71 | 5604 |
|  |  | 442Luxembourg | 2.42 | .69 | 4559 |
|  |  | 446Macao | 2.62 | .67 | 3740 |
|  |  | 458Malaysia | 2.77 | .60 | 5815 |
|  |  | 470Malta | 2.81 | .69 | 2793 |
|  |  | 484Mexico | 2.48 | .64 | 4145 |
|  |  | 498Moldova | 2.57 | .58 | 4931 |
|  |  | 499Montenegro | 2.69 | .74 | 5526 |
|  |  | 504Morocco | 2.67 | .67 | 2779 |
|  |  | 528Netherlands | 2.20 | .59 | 3633 |
|  |  | 554New Zealand | 2.79 | .67 | 5126 |
|  |  | 591Panama | 2.55 | .75 | 2033 |
|  |  | 604Peru | 2.63 | .58 | 2508 |
|  |  | 608Philippines | 2.67 | .65 | 6438 |
|  |  | 616Poland | 2.67 | .63 | 5073 |
|  |  | 620Portugal | 2.66 | .69 | 4982 |
|  |  | 634Qatar | 2.65 | .73 | 11579 |
|  |  | 642Romania | 2.59 | .65 | 4575 |
|  |  | 643Russian Federation | 2.44 | .69 | 6406 |
|  |  | 682Saudi Arabia | 2.64 | .76 | 5390 |
|  |  | 688Serbia | 2.48 | .76 | 4885 |
|  |  | 702Singapore | 2.99 | .65 | 6429 |
|  |  | 703Slovak Republic | 2.44 | .66 | 4853 |
|  |  | 704Vietnam | 2.32 | .65 | 5325 |
|  |  | 705Slovenia | 2.37 | .69 | 5136 |
|  |  | 724Spain | 2.47 | .72 | 27259 |
|  |  | 752Sweden | 2.61 | .73 | 4827 |
|  |  | 756Switzerland | 2.41 | .69 | 3732 |
|  |  | 764Thailand | 2.62 | .64 | 8313 |
|  |  | 784United Arab Emirates | 2.66 | .74 | 17119 |
|  |  | 792Turkey | 2.75 | .79 | 6560 |
|  |  | 804Ukraine | 2.34 | .68 | 5221 |
|  |  | 826United Kingdom | 2.77 | .65 | 12157 |
|  |  | 840United States | 2.81 | .70 | 4531 |
|  |  | 858Uruguay | 2.34 | .77 | 3041 |
|  |  | 975B-S-J-Z (China) | 2.57 | .69 | 11905 |
|  |  | 982Moscow Region (RUS) | 2.40 | .70 | 1727 |
|  |  | 983Tatarstan (RUS) | 2.44 | .69 | 5084 |
| Peer Cooperation |  | 8Albania | 3.14 | .66 | 5898 |
|  |  | 31Baku (Azerbaijan) | 2.74 | .78 | 3511 |
|  |  | 32Argentina | 2.32 | .71 | 7748 |
|  |  | 36Australia | 2.69 | .66 | 10374 |
|  |  | 40Austria | 2.92 | .74 | 5132 |
|  |  | 56Belgium | 2.62 | .67 | 6982 |
|  |  | 70Bosnia and Herzegovina | 2.71 | .76 | 5356 |
|  |  | 76Brazil | 2.40 | .75 | 6171 |
|  |  | 96Brunei Darussalam | 2.65 | .66 | 4538 |
|  |  | 100Bulgaria | 2.42 | .78 | 3614 |
|  |  | 112Belarus | 2.57 | .71 | 5319 |
|  |  | 152Chile | 2.58 | .74 | 4847 |
|  |  | 158Chinese Taipei | 2.85 | .74 | 7026 |
|  |  | 170Colombia | 2.58 | .70 | 5106 |
|  |  | 188Costa Rica | 2.54 | .76 | 6190 |
|  |  | 191Croatia | 2.66 | .74 | 5336 |
|  |  | 203Czech Republic | 2.52 | .68 | 6039 |
|  |  | 208Denmark | 2.87 | .63 | 5863 |
|  |  | 214Dominican Republic | 2.64 | .80 | 1390 |
|  |  | 233Estonia | 2.58 | .67 | 4747 |
|  |  | 246Finland | 2.71 | .63 | 4904 |
|  |  | 250France | 2.40 | .71 | 4482 |
|  |  | 268Georgia | 2.84 | .80 | 4225 |
|  |  | 276Germany | 2.70 | .73 | 2149 |
|  |  | 300Greece | 2.67 | .72 | 5336 |
|  |  | 344Hong Kong | 2.70 | .66 | 5552 |
|  |  | 348Hungary | 2.58 | .71 | 4191 |
|  |  | 352Iceland | 2.87 | .71 | 2317 |
|  |  | 360Indonesia | 2.91 | .63 | 11489 |
|  |  | 372Ireland | 2.56 | .65 | 4328 |
|  |  | 380Italy | 2.48 | .69 | 8494 |
|  |  | 383Kosovo | 3.02 | .71 | 4254 |
|  |  | 392Japan | 2.73 | .76 | 5833 |
|  |  | 398Kazakhstan | 2.86 | .72 | 15384 |
|  |  | 400Jordan | 2.72 | .80 | 7915 |
|  |  | 410Korea | 2.78 | .75 | 6547 |
|  |  | 428Latvia | 2.51 | .65 | 4507 |
|  |  | 440Lithuania | 2.81 | .76 | 5461 |
|  |  | 442Luxembourg | 2.62 | .73 | 4424 |
|  |  | 446Macao | 2.72 | .65 | 3736 |
|  |  | 458Malaysia | 3.06 | .65 | 5732 |
|  |  | 470Malta | 2.62 | .69 | 2699 |
|  |  | 484Mexico | 2.65 | .71 | 3721 |
|  |  | 498Moldova | 2.77 | .63 | 4889 |
|  |  | 499Montenegro | 2.74 | .77 | 5440 |
|  |  | 504Morocco | 2.56 | .75 | 2428 |
|  |  | 528Netherlands | 2.75 | .60 | 3554 |
|  |  | 554New Zealand | 2.66 | .65 | 4879 |
|  |  | 591Panama | 2.60 | .76 | 1752 |
|  |  | 604Peru | 2.70 | .66 | 2010 |
|  |  | 608Philippines | 2.80 | .68 | 6155 |
|  |  | 616Poland | 2.66 | .68 | 4937 |
|  |  | 620Portugal | 2.62 | .66 | 4756 |
|  |  | 634Qatar | 2.67 | .78 | 11350 |
|  |  | 642Romania | 2.75 | .67 | 4439 |
|  |  | 643Russian Federation | 2.62 | .71 | 6320 |
|  |  | 682Saudi Arabia | 2.60 | .83 | 5319 |
|  |  | 688Serbia | 2.60 | .77 | 4745 |
|  |  | 702Singapore | 2.83 | .66 | 6330 |
|  |  | 703Slovak Republic | 2.52 | .71 | 4694 |
|  |  | 704Vietnam | 2.66 | .60 | 5293 |
|  |  | 705Slovenia | 2.63 | .70 | 4835 |
|  |  | 724Spain | 2.61 | .72 | 25984 |
|  |  | 752Sweden | 2.62 | .70 | 4667 |
|  |  | 756Switzerland | 2.75 | .73 | 3378 |
|  |  | 764Thailand | 2.81 | .66 | 8235 |
|  |  | 784United Arab Emirates | 2.80 | .81 | 16851 |
|  |  | 792Turkey | 2.66 | .85 | 6520 |
|  |  | 804Ukraine | 2.73 | .74 | 5012 |
|  |  | 826United Kingdom | 2.59 | .65 | 11708 |
|  |  | 840United States | 2.55 | .68 | 4474 |
|  |  | 858Uruguay | 2.50 | .74 | 2737 |
|  |  | 975B-S-J-Z (China) | 2.85 | .73 | 11898 |
|  |  | 982Moscow Region (RUS) | 2.56 | .74 | 1696 |
|  |  | 983Tatarstan (RUS) | 2.63 | .72 | 5035 |
| Fear of Failure |  | 8Albania | 2.39 | .80 | 6045 |
|  |  | 31Baku (Azerbaijan) | 2.62 | .86 | 5108 |
|  |  | 32Argentina | 2.45 | .81 | 10516 |
|  |  | 36Australia | 2.78 | .79 | 12007 |
|  |  | 40Austria | 2.36 | .85 | 6379 |
|  |  | 56Belgium | 2.44 | .73 | 4514 |
|  |  | 70Bosnia and Herzegovina | 2.28 | .79 | 5875 |
|  |  | 76Brazil | 2.59 | .81 | 8698 |
|  |  | 96Brunei Darussalam | 2.93 | .75 | 6435 |
|  |  | 100Bulgaria | 2.47 | .82 | 4376 |
|  |  | 112Belarus | 2.48 | .65 | 5657 |
|  |  | 152Chile | 2.63 | .80 | 6711 |
|  |  | 158Chinese Taipei | 3.11 | .68 | 7112 |
|  |  | 170Colombia | 2.40 | .78 | 6694 |
|  |  | 188Costa Rica | 2.37 | .82 | 6418 |
|  |  | 191Croatia | 2.40 | .78 | 6313 |
|  |  | 203Czech Republic | 2.58 | .73 | 6554 |
|  |  | 208Denmark | 2.54 | .75 | 6598 |
|  |  | 214Dominican Republic | 2.56 | .85 | 3431 |
|  |  | 233Estonia | 2.43 | .75 | 5108 |
|  |  | 246Finland | 2.43 | .78 | 5321 |
|  |  | 250France | 2.60 | .85 | 5801 |
|  |  | 268Georgia | 2.32 | .72 | 4750 |
|  |  | 276Germany | 2.28 | .83 | 4195 |
|  |  | 300Greece | 2.51 | .77 | 6067 |
|  |  | 344Hong Kong | 2.94 | .67 | 5705 |
|  |  | 348Hungary | 2.50 | .82 | 4926 |
|  |  | 352Iceland | 2.59 | .86 | 3024 |
|  |  | 360Indonesia | 2.47 | .73 | 11712 |
|  |  | 372Ireland | 2.75 | .78 | 5408 |
|  |  | 380Italy | 2.56 | .79 | 10768 |
|  |  | 383Kosovo | 2.53 | .76 | 4619 |
|  |  | 392Japan | 2.88 | .75 | 6008 |
|  |  | 398Kazakhstan | 2.32 | .72 | 18388 |
|  |  | 400Jordan | 2.41 | .84 | 8527 |
|  |  | 410Korea | 2.74 | .76 | 6590 |
|  |  | 428Latvia | 2.51 | .72 | 5006 |
|  |  | 440Lithuania | 2.52 | .83 | 6457 |
|  |  | 442Luxembourg | 2.46 | .85 | 4881 |
|  |  | 446Macao | 2.95 | .67 | 3763 |
|  |  | 458Malaysia | 2.88 | .71 | 6010 |
|  |  | 470Malta | 2.77 | .80 | 3100 |
|  |  | 484Mexico | 2.61 | .77 | 5763 |
|  |  | 498Moldova | 2.57 | .67 | 5204 |
|  |  | 499Montenegro | 2.22 | .80 | 6093 |
|  |  | 504Morocco | 2.44 | .81 | 4978 |
|  |  | 528Netherlands | 2.27 | .75 | 3779 |
|  |  | 554New Zealand | 2.80 | .80 | 5889 |
|  |  | 591Panama | 2.51 | .83 | 3986 |
|  |  | 604Peru | 2.41 | .74 | 4669 |
|  |  | 608Philippines | 2.74 | .70 | 6973 |
|  |  | 616Poland | 2.60 | .74 | 5472 |
|  |  | 620Portugal | 2.58 | .78 | 5513 |
|  |  | 634Qatar | 2.53 | .86 | 12030 |
|  |  | 642Romania | 2.34 | .74 | 4916 |
|  |  | 643Russian Federation | 2.47 | .73 | 6984 |
|  |  | 682Saudi Arabia | 2.30 | .85 | 5818 |
|  |  | 688Serbia | 2.33 | .78 | 5714 |
|  |  | 702Singapore | 2.99 | .76 | 6595 |
|  |  | 703Slovak Republic | 2.59 | .72 | 5476 |
|  |  | 704Vietnam | 2.60 | .63 | 5363 |
|  |  | 705Slovenia | 2.58 | .77 | 5968 |
|  |  | 724Spain | 2.49 | .78 | 33517 |
|  |  | 752Sweden | 2.57 | .81 | 5095 |
|  |  | 756Switzerland | 2.37 | .81 | 5217 |
|  |  | 764Thailand | 2.74 | .69 | 8467 |
|  |  | 784United Arab Emirates | 2.63 | .83 | 17589 |
|  |  | 792Turkey | 2.70 | .80 | 6745 |
|  |  | 804Ukraine | 2.40 | .64 | 5852 |
|  |  | 826United Kingdom | 2.81 | .81 | 12838 |
|  |  | 840United States | 2.69 | .86 | 4626 |
|  |  | 858Uruguay | 2.49 | .80 | 4188 |
|  |  | 975B-S-J-Z (China) | 2.65 | .69 | 11964 |
|  |  | 982Moscow Region (RUS) | 2.45 | .72 | 1881 |
|  |  | 983Tatarstan (RUS) | 2.47 | .72 | 5390 |
| Work Mastery |  | 8Albania | 3.33 | .56 | 5914 |
|  |  | 31Baku (Azerbaijan) | 3.04 | .76 | 4964 |
|  |  | 32Argentina | 3.14 | .61 | 10444 |
|  |  | 36Australia | 2.91 | .56 | 11919 |
|  |  | 40Austria | 2.93 | .62 | 6355 |
|  |  | 56Belgium | 2.74 | .49 | 4464 |
|  |  | 70Bosnia and Herzegovina | 3.03 | .62 | 5739 |
|  |  | 76Brazil | 3.13 | .58 | 8626 |
|  |  | 96Brunei Darussalam | 3.05 | .49 | 6387 |
|  |  | 100Bulgaria | 3.00 | .68 | 4282 |
|  |  | 112Belarus | 2.97 | .51 | 5591 |
|  |  | 152Chile | 3.14 | .60 | 6664 |
|  |  | 158Chinese Taipei | 3.08 | .50 | 7080 |
|  |  | 170Colombia | 3.10 | .57 | 6622 |
|  |  | 188Costa Rica | 3.29 | .66 | 6348 |
|  |  | 191Croatia | 3.08 | .57 | 6258 |
|  |  | 203Czech Republic | 2.80 | .54 | 6491 |
|  |  | 208Denmark | 2.94 | .55 | 6558 |
|  |  | 214Dominican Republic | 3.08 | .74 | 3381 |
|  |  | 233Estonia | 2.81 | .53 | 5051 |
|  |  | 246Finland | 2.77 | .59 | 5242 |
|  |  | 250France | 2.84 | .62 | 5618 |
|  |  | 268Georgia | 3.05 | .64 | 4551 |
|  |  | 276Germany | 2.89 | .58 | 4176 |
|  |  | 300Greece | 3.14 | .56 | 6002 |
|  |  | 344Hong Kong | 2.93 | .50 | 5687 |
|  |  | 348Hungary | 2.97 | .55 | 4901 |
|  |  | 352Iceland | 2.89 | .64 | 2961 |
|  |  | 360Indonesia | 3.16 | .54 | 11624 |
|  |  | 372Ireland | 2.88 | .53 | 5341 |
|  |  | 380Italy | 3.14 | .60 | 10682 |
|  |  | 383Kosovo | 3.19 | .58 | 4453 |
|  |  | 392Japan | 2.82 | .64 | 5986 |
|  |  | 398Kazakhstan | 2.97 | .68 | 18231 |
|  |  | 400Jordan | 3.20 | .68 | 8489 |
|  |  | 410Korea | 3.16 | .51 | 6569 |
|  |  | 428Latvia | 2.89 | .57 | 4971 |
|  |  | 440Lithuania | 2.93 | .63 | 6413 |
|  |  | 442Luxembourg | 2.80 | .64 | 4804 |
|  |  | 446Macao | 2.94 | .49 | 3744 |
|  |  | 458Malaysia | 3.23 | .51 | 5996 |
|  |  | 470Malta | 3.09 | .59 | 3075 |
|  |  | 484Mexico | 3.20 | .63 | 5750 |
|  |  | 498Moldova | 3.00 | .49 | 5147 |
|  |  | 499Montenegro | 2.98 | .64 | 5925 |
|  |  | 504Morocco | 3.15 | .59 | 4841 |
|  |  | 528Netherlands | 2.73 | .51 | 3748 |
|  |  | 554New Zealand | 2.91 | .56 | 5833 |
|  |  | 591Panama | 3.22 | .66 | 3953 |
|  |  | 604Peru | 3.22 | .59 | 4677 |
|  |  | 608Philippines | 3.03 | .59 | 6884 |
|  |  | 616Poland | 2.98 | .54 | 5442 |
|  |  | 620Portugal | 3.07 | .53 | 5438 |
|  |  | 634Qatar | 3.12 | .68 | 11857 |
|  |  | 642Romania | 3.01 | .54 | 4888 |
|  |  | 643Russian Federation | 2.82 | .55 | 6924 |
|  |  | 682Saudi Arabia | 3.09 | .65 | 5783 |
|  |  | 688Serbia | 2.99 | .62 | 5616 |
|  |  | 702Singapore | 3.06 | .51 | 6568 |
|  |  | 703Slovak Republic | 2.87 | .56 | 5388 |
|  |  | 704Vietnam | 3.06 | .45 | 5355 |
|  |  | 705Slovenia | 3.11 | .57 | 5948 |
|  |  | 724Spain | 3.16 | .55 | 33234 |
|  |  | 752Sweden | 2.79 | .62 | 5028 |
|  |  | 756Switzerland | 2.87 | .59 | 5189 |
|  |  | 764Thailand | 3.15 | .50 | 8424 |
|  |  | 784United Arab Emirates | 3.16 | .67 | 17379 |
|  |  | 792Turkey | 2.89 | .65 | 6689 |
|  |  | 804Ukraine | 2.94 | .54 | 5832 |
|  |  | 826United Kingdom | 2.82 | .57 | 12533 |
|  |  | 840United States | 3.04 | .58 | 4580 |
|  |  | 858Uruguay | 3.12 | .66 | 4126 |
|  |  | 975B-S-J-Z (China) | 3.14 | .50 | 11931 |
|  |  | 982Moscow Region (RUS) | 2.81 | .57 | 1876 |
|  |  | 983Tatarstan (RUS) | 2.86 | .56 | 5346 |
| ESCS |  | 8Albania | -.83 | .96 | 6277 |
|  |  | 31Baku (Azerbaijan) | -.54 | .92 | 6597 |
|  |  | 32Argentina | -.80 | 1.19 | 11845 |
|  |  | 36Australia | .32 | .91 | 12813 |
|  |  | 40Austria | .04 | .87 | 6674 |
|  |  | 56Belgium | .10 | .92 | 8312 |
|  |  | 70Bosnia and Herzegovina | -.56 | .82 | 6379 |
|  |  | 76Brazil | -1.13 | 1.22 | 10453 |
|  |  | 96Brunei Darussalam | -.26 | .97 | 6790 |
|  |  | 100Bulgaria | -.22 | 1.01 | 5113 |
|  |  | 112Belarus | -.11 | .76 | 5763 |
|  |  | 152Chile | -.31 | 1.10 | 7411 |
|  |  | 158Chinese Taipei | -.33 | .92 | 7172 |
|  |  | 170Colombia | -1.06 | 1.26 | 7201 |
|  |  | 188Costa Rica | -.98 | 1.29 | 7182 |
|  |  | 191Croatia | -.24 | .78 | 6576 |
|  |  | 203Czech Republic | -.09 | .88 | 6911 |
|  |  | 208Denmark | .39 | .84 | 7431 |
|  |  | 214Dominican Republic | -1.05 | 1.12 | 5534 |
|  |  | 233Estonia | .10 | .80 | 5202 |
|  |  | 246Finland | .30 | .79 | 5557 |
|  |  | 250France | -.11 | .97 | 6176 |
|  |  | 268Georgia | -.37 | .91 | 5472 |
|  |  | 276Germany | -.09 | 1.04 | 4656 |
|  |  | 300Greece | -.09 | .91 | 6372 |
|  |  | 344Hong Kong | -.52 | 1.02 | 5839 |
|  |  | 348Hungary | -.06 | .92 | 5083 |
|  |  | 352Iceland | .54 | .82 | 3222 |
|  |  | 360Indonesia | -1.37 | 1.11 | 12008 |
|  |  | 372Ireland | .13 | .86 | 5519 |
|  |  | 380Italy | -.22 | .89 | 11475 |
|  |  | 383Kosovo | -.47 | .88 | 5003 |
|  |  | 392Japan | -.11 | .73 | 6055 |
|  |  | 398Kazakhstan | -.33 | .86 | 19451 |
|  |  | 400Jordan | -.72 | 1.13 | 8902 |
|  |  | 410Korea | .09 | .77 | 6626 |
|  |  | 428Latvia | -.01 | .82 | 5187 |
|  |  | 440Lithuania | .03 | .86 | 6693 |
|  |  | 442Luxembourg | .01 | 1.15 | 5082 |
|  |  | 446Macao | -.52 | .91 | 3766 |
|  |  | 458Malaysia | -.75 | 1.05 | 6040 |
|  |  | 470Malta | .08 | .95 | 3295 |
|  |  | 484Mexico | -1.07 | 1.23 | 6475 |
|  |  | 498Moldova | -.58 | .93 | 5340 |
|  |  | 499Montenegro | -.17 | .87 | 6607 |
|  |  | 504Morocco | -1.91 | 1.42 | 6731 |
|  |  | 528Netherlands | .26 | .88 | 4659 |
|  |  | 554New Zealand | .18 | .97 | 6013 |
|  |  | 591Panama | -1.03 | 1.33 | 6088 |
|  |  | 604Peru | -1.10 | 1.16 | 6042 |
|  |  | 608Philippines | -1.44 | 1.11 | 7188 |
|  |  | 616Poland | -.14 | .85 | 5556 |
|  |  | 620Portugal | -.37 | 1.15 | 5632 |
|  |  | 634Qatar | .28 | .84 | 13450 |
|  |  | 642Romania | -.46 | .95 | 5056 |
|  |  | 643Russian Federation | .17 | .73 | 7438 |
|  |  | 682Saudi Arabia | -.66 | 1.18 | 6086 |
|  |  | 688Serbia | -.25 | .83 | 6516 |
|  |  | 702Singapore | .15 | .92 | 6648 |
|  |  | 703Slovak Republic | -.18 | .92 | 5884 |
|  |  | 704Vietnam | -1.61 | 1.08 | 5376 |
|  |  | 705Slovenia | -.01 | .79 | 6331 |
|  |  | 724Spain | -.04 | 1.03 | 35290 |
|  |  | 752Sweden | .36 | .89 | 5348 |
|  |  | 756Switzerland | .00 | .92 | 5705 |
|  |  | 764Thailand | -1.05 | 1.25 | 8582 |
|  |  | 784United Arab Emirates | .23 | .91 | 18751 |
|  |  | 792Turkey | -1.17 | 1.18 | 6855 |
|  |  | 804Ukraine | -.19 | .77 | 5971 |
|  |  | 826United Kingdom | .24 | .89 | 12893 |
|  |  | 840United States | .06 | 1.01 | 4767 |
|  |  | 858Uruguay | -.98 | 1.17 | 5058 |
|  |  | 975B-S-J-Z (China) | -.36 | 1.09 | 11990 |
|  |  | 982Moscow Region (RUS) | .35 | .68 | 1995 |
|  |  | 983Tatarstan (RUS) | .17 | .71 | 5693 |

**Table S2**

*Correlations among the Focal Variables across Countries*

|  |  | Correlation with Mastery-approach Goals | | |
| --- | --- | --- | --- | --- |
|  |  | Individual Level | | School Level |
| Overall (*n*_student_ = 6359, *n*_school_ = 327) | | | | |
|  | Peer Competition | .201** | | .134** |
|  | Peer Cooperation | .282** | | .143** |
|  | Fear of Failure | .048** | | - |
|  | Work Mastery | .422** | | - |
|  | Gender | -.095** | | - |
|  | Grade | -.016** | | - |
|  | ESCS | .034** | | -.034** |
|  | School Type | - | | -.013** |
|  | School Size | - | | .024** |
| 8Albania (*n*_student_ = 6359, *n*_school_ = 327) | | | | |
|  | Peer Competition | .304** | | .142** |
|  | Peer Cooperation | .382** | | .181** |
|  | Fear of Failure | .023 | | - |
|  | Work Mastery | .452** | | - |
|  | Gender | -.230** | | - |
|  | Grade | .007 | | - |
|  | ESCS | .070** | | -.006 |
|  | School Type | - | | -.008 |
|  | School Size | - | | .020 |
| 31Baku (Azerbaijan) (*n*_student_ = 6827, *n*_school_ = 197) | | | | |
|  | Peer Competition | .290** | | .075** |
|  | Peer Cooperation | .321** | | .096** |
|  | Fear of Failure | .107** | | - |
|  | Work Mastery | .386** | | - |
|  | Gender | -.119** | | - |
|  | Grade | .051** | | - |
|  | ESCS | .084** | | -.009 |
|  | School Type | - | | -.018 |
|  | School Size | - | | -.030 |
| 32Argentina (*n*_student_ = 11975, *n*_school_ = 455) | | | | |
|  | Peer Competition | .165** | | .045** |
|  | Peer Cooperation | .263** | | .048** |
|  | Fear of Failure | .054** | | - |
|  | Work Mastery | .372** | | - |
|  | Gender | -.017 | | - |
|  | Grade | .008 | | - |
|  | ESCS | .013 | | -.060** |
|  | School Type | - | | .049** |
|  | School Size | - | | -.010 |
| 36Australia (*n*_student_ = 14273, *n*_school_ = 763) | | | | |
|  | Peer Competition | .179** | | .092** |
|  | Peer Cooperation | .282** | | .121** |
|  | Fear of Failure | .129** | | .129** |
|  | Work Mastery | .552** | | - |
|  | Gender | -.114** | | - |
|  | Grade | .008 | | - |
|  | ESCS | .168** | | .095** |
|  | School Type | - | | -.069** |
|  | School Size | - | | .043** |
| 40Austria (*n*_student_ = 6802, *n*_school_ = 291) | | | | |
|  | Peer Competition | .156** | | .044** |
|  | Peer Cooperation | .224** | | .104** |
|  | Fear of Failure | .078** | | - |
|  | Work Mastery | .457** | | - |
|  | Gender | -.086** | | - |
|  | Grade | - | | - |
|  | ESCS | .005 | | -.001 |
|  | School Type | - | | -.005 |
|  | School Size | - | | - |
| 56Belgium (*n*_student_ = 8475, *n*_school_ = 288) | | | | |
|  | Peer Competition | .003 | | -.207** |
|  | Peer Cooperation | .051** | | -.231** |
|  | Fear of Failure | .221** | | - |
|  | Work Mastery | .486** | | - |
|  | Gender | -.161** | | - |
|  | Grade | -.044** | | - |
|  | ESCS | -.010 | | -.051** |
|  | School Type | - | | - |
|  | School Size | - | | .038** |
| 70Bosnia and Herzegovina (*n*_student_ = 6480, *n*_school_ = 213) | | | | |
|  | Peer Competition | .249** | | .107** |
|  | Peer Cooperation | .287** | | .121** |
|  | Fear of Failure | .033* | | - |
|  | Work Mastery | .388** | | - |
|  | Gender | -.109** | | - |
|  | Grade | -.007 | | - |
|  | ESCS | .052** | | .044** |
|  | School Type | - | | -.028* |
|  | School Size | - | | -.007 |
| 76Brazil (*n*_student_ = 10691, *n*_school_ = 597) | | | | |
|  | Peer Competition | .114** | | .070** |
|  | Peer Cooperation | .204** | | .079** |
|  | Fear of Failure | .009 | | - |
|  | Work Mastery | .466** | | - |
|  | Gender | -.126** | | - |
|  | Grade | .120** | | - |
|  | ESCS | .020 | | .020 |
|  | School Type | - | | .006 |
|  | School Size | - | | .021 |
| 96Brunei Darussalam (*n*_student_ = 6828, *n*_school_ = 55) | | | | |
|  | Peer Competition | .299** | | .085** |
|  | Peer Cooperation | .279** | | .075** |
|  | Fear of Failure | .120** | | - |
|  | Work Mastery | .360** | | - |
|  | Gender | -.095** | | - |
|  | Grade | .084** | | - |
|  | ESCS | .115** | | .049** |
|  | School Type | - | | .054** |
|  | School Size | - | | -.018 |
| 100Bulgaria (*n*_student_ = 5294, *n*_school_ = 197) | | | | |
|  | Peer Competition | .210** | | .062** |
|  | Peer Cooperation | .289** | | .099** |
|  | Fear of Failure | .128** | | - |
|  | Work Mastery | .449** | | - |
|  | Gender | -.088** | | - |
|  | Grade | -.002 | | - |
|  | ESCS | .084** | | .046** |
|  | School Type | - | | .000 |
|  | School Size | - | | -.001 |
| 112Belarus (*n*_student_ = 5803, *n*_school_ = 234) | | | | |
|  | Peer Competition | .158** | | .052** |
|  | Peer Cooperation | .260** | | .119** |
|  | Fear of Failure | .039** | | - |
|  | Work Mastery | .393** | | - |
|  | Gender | -.068** | | - |
|  | Grade | -.010 | | - |
|  | ESCS | .084** | | -.002 |
|  | School Type | - | | .012 |
|  | School Size | - | | -.061** |
| 152Chile (*n*_student_ = 7621, *n*_school_ = 254) | | | | |
|  | Peer Competition | .127** | | .023 |
|  | Peer Cooperation | .231** | | .059** |
|  | Fear of Failure | .029* | | - |
|  | Work Mastery | .431** | | - |
|  | Gender | -.098** | | - |
|  | Grade | .016 | | - |
|  | ESCS | .020 | | -.020 |
|  | School Type | - | | -.010 |
|  | School Size | - | | -.009 |
| 158Chinese Taipei (*n*_student_ = 7243, *n*_school_ = 192) | | | | |
|  | Peer Competition | .222** | | .112** |
|  | Peer Cooperation | .268** | | .125** |
|  | Fear of Failure | .099** | | - |
|  | Work Mastery | .420** | | - |
|  | Gender | -.052** | | - |
|  | Grade | -.010 | | - |
|  | ESCS | .147** | | .097** |
|  | School Type | - | | .024* |
|  | School Size | - | | -.004 |
| 170Colombia (*n*_student_ = 7522, *n*_school_ = 247) | | | | |
|  | Peer Competition | .180** | | .030* |
|  | Peer Cooperation | .206** | | .050** |
|  | Fear of Failure | 0.011 | | - |
|  | Work Mastery | .352** | | - |
|  | Gender | -.080** | | - |
|  | Grade | .065** | | - |
|  | ESCS | .028* | | -.010 |
|  | School Type | - | | .026* |
|  | School Size | - | | .005 |
| 188Costa Rica (*n*_student_ = 7221, *n*_school_ = 205) | | | | |
|  | Peer Competition | .164** | | 0.009 |
|  | Peer Cooperation | .252** | | .054** |
|  | Fear of Failure | -.006 | | - |
|  | Work Mastery | .362** | | - |
|  | Gender | -.062** | | - |
|  | Grade | .058** | | - |
|  | ESCS | -.020 | | -.051** |
|  | School Type | - | | .045** |
|  | School Size | - | | -.012 |
| 191Croatia (*n*_student_ = 6609, *n*_school_ = 183) | | | | |
|  | Peer Competition | .018 | | .015 |
|  | Peer Cooperation | .296** | | .107** |
|  | Fear of Failure | .061** | | - |
|  | Work Mastery | .405** | | - |
|  | Gender | -.055** | | - |
|  | Grade | -.037** | | - |
|  | ESCS | .068** | | .048** |
|  | School Type | - | | .012 |
|  | School Size | - | | .038** |
| 203Czech Republic (*n*_student_ = 7019, *n*_school_ = 333) | | | | |
|  | Peer Competition | .062** | | -.029* |
|  | Peer Cooperation | .233** | | .120** |
|  | Fear of Failure | .129** | | - |
|  | Work Mastery | .387** | | - |
|  | Gender | -.102** | | - |
|  | Grade | .053** | | - |
|  | ESCS | .106** | | .081** |
|  | School Type | - | | -.013 |
|  | School Size | - | | .016 |
| 208Denmark (*n*_student_ = 7657, *n*_school_ = 348) | | | | |
|  | Peer Competition | .117** | | .047** |
|  | Peer Cooperation | .253** | | .082** |
|  | Fear of Failure | .086** | | - |
|  | Work Mastery | .517** | | - |
|  | Gender | -.163** | | - |
|  | Grade | .002 | | - |
|  | ESCS | .077** | | -.026* |
|  | School Type | - | | -.014 |
|  | School Size | - | | -.008 |
| 214Dominican Republic (*n*_student_ = 5674, *n*_school_ = 235) | | | | |
|  | Peer Competition | .250** | | .060** |
|  | Peer Cooperation | .275** | | .049** |
|  | Fear of Failure | .106** | | - |
|  | Work Mastery | .383** | | - |
|  | Gender | -.080** | | - |
|  | Grade | .158** | | - |
|  | ESCS | .002 | | -.044* |
|  | School Type | - | | .060** |
|  | School Size | - | | .003 |
| 233Estonia (*n*_student_ = 5316, *n*_school_ = 230) | | | | |
|  | Peer Competition | .190** | | .152** |
|  | Peer Cooperation | .276** | | .187** |
|  | Fear of Failure | .059** | | - |
|  | Work Mastery | .373** | | - |
|  | Gender | -.055** | | - |
|  | Grade | .000 | | - |
|  | ESCS | .167** | | .120** |
|  | School Type | - | | -.032* |
|  | School Size | - | | .011 |
| 246Finland (*n*_student_ = 5649, *n*_school_ = 214) | | | | |
|  | Peer Competition | .186** | | .086** |
|  | Peer Cooperation | .312** | | .099** |
|  | Fear of Failure | .058** | | - |
|  | Work Mastery | .510** | | - |
|  | Gender | -.070** | | - |
|  | Grade | -.016 | | - |
|  | ESCS | .191** | | .103** |
|  | School Type | - | | -.037** |
|  | School Size | - | | .048** |
| 250France (*n*_student_ = 6308, *n*_school_ = 252) | | | | |
|  | Peer Competition | .111** | | .058** |
|  | Peer Cooperation | .197** | | .046** |
|  | Fear of Failure | .154** | | - |
|  | Work Mastery | .526** | | - |
|  | Gender | -.110** | | - |
|  | Grade | .018 | | - |
|  | ESCS | .089** | | .003 |
|  | School Type | - | | -.024 |
|  | School Size | - | | .001 |
| 268Georgia (*n*_student_ = 5572, *n*_school_ = 321) | | | | |
|  | Peer Competition | .108** | | -.013 |
|  | Peer Cooperation | .271** | | .115** |
|  | Fear of Failure | .059** | | - |
|  | Work Mastery | .411** | | - |
|  | Gender | -.229** | | - |
|  | Grade | .038* | | - |
|  | ESCS | .103** | | .001 |
|  | School Type | - | | -.042** |
|  | School Size | - | | -.055** |
| 276Germany (*n*_student_ = 5451, *n*_school_ = 223) | | | | |
|  | Peer Competition | .141** | | .021 |
|  | Peer Cooperation | .207** | | .055** |
|  | Fear of Failure | .084** | | - |
|  | Work Mastery | .441** | | - |
|  | Gender | -.144** | | - |
|  | Grade | .023 | | - |
|  | ESCS | .042** | | .019 |
|  | School Type | - | | -.010 |
|  | School Size | - | | .029 |
| 300Greece (*n*_student_ = 6403, *n*_school_ = 242) | | | | |
|  | Peer Competition | .117** | | .093** |
|  | Peer Cooperation | .234** | | .108** |
|  | Fear of Failure | .047** | | - |
|  | Work Mastery | .430** | | - |
|  | Gender | -.100** | | - |
|  | Grade | .040** | | - |
|  | ESCS | .136** | | .092** |
|  | School Type | - | | -.056** |
|  | School Size | - | | -.013 |
| 344Hong Kong (*n*_student_ = 6037, *n*_school_ = 152) | | | | |
|  | Peer Competition | .174** | | .099** |
|  | Peer Cooperation | .293** | | .138** |
|  | Fear of Failure | .108** | | - |
|  | Work Mastery | .388** | | - |
|  | Gender | -.060** | | - |
|  | Grade | .024 | | - |
|  | ESCS | .134** | | .122** |
|  | School Type | - | | .010 |
|  | School Size | - | | .063** |
| 348Hungary (*n*_student_ = 5132, *n*_school_ = 238) | | | | |
|  | Peer Competition | .145** | | .062** |
|  | Peer Cooperation | .307** | | .170** |
|  | Fear of Failure | .053** | | - |
|  | Work Mastery | .422** | | - |
|  | Gender | -.041** | | - |
|  | Grade | .034* | | - |
|  | ESCS | .116** | | .124** |
|  | School Type | - | | -.022 |
|  | School Size | - | | .061** |
| 352Iceland (*n*_student_ = 3296, *n*_school_ = 142) | | | | |
|  | Peer Competition | .122** | | .074** |
|  | Peer Cooperation | .314** | | .112** |
|  | Fear of Failure | .059** | | - |
|  | Work Mastery | .464** | | - |
|  | Gender | -.191** | | - |
|  | Grade | .016 | | - |
|  | ESCS | .193** | | .075** |
|  | School Type | - | | -.011 |
|  | School Size | - | | .043* |
| 360Indonesia (*n*_student_ = 12098, *n*_school_ = 397) | | | | |
|  | Peer Competition | .190** | | .031** |
|  | Peer Cooperation | .331** | | .113** |
|  | Fear of Failure | -.027** | | - |
|  | Work Mastery | .332** | | - |
|  | Gender | -.085** | | - |
|  | Grade | -.009 | | - |
|  | ESCS | -.056** | | -.075** |
|  | School Type | - | | .014 |
|  | School Size | - | | -.005 |
| 372Ireland (*n*_student_ = 5577, *n*_school_ = 157) | | | | |
|  | Peer Competition | .123** | | .027* |
|  | Peer Cooperation | .270** | | .092** |
|  | Fear of Failure | .084** | | - |
|  | Work Mastery | .462** | | - |
|  | Gender | -.148** | | - |
|  | Grade | -.052** | | - |
|  | ESCS | .180** | | .097** |
|  | School Type | - | | - |
|  | School Size | - | | .010 |
| 380Italy (*n*_student_ = 11785, *n*_school_ = 542) | | | | |
|  | Peer Competition | .158** | | .085** |
|  | Peer Cooperation | .217** | | .054** |
|  | Fear of Failure | .042** | | - |
|  | Work Mastery | .383** | | - |
|  | Gender | -.075** | | - |
|  | Grade | .082** | | - |
|  | ESCS | .092** | | .074** |
|  | School Type | - | | -.007 |
|  | School Size | - | | .026** |
| 383Kosovo (*n*_student_ = 5058, *n*_school_ = 211) | | | | |
|  | Peer Competition | .307** | | .100** |
|  | Peer Cooperation | .395** | | .181** |
|  | Fear of Failure | .103** | | - |
|  | Work Mastery | .495** | | - |
|  | Gender | -.194** | | - |
|  | Grade | -.006 | | - |
|  | ESCS | .055** | | .025 |
|  | School Type | - | | .002 |
|  | School Size | - | | -.007 |
| 392Japan (*n*_student_ = 6109, *n*_school_ = 183) | | | | |
|  | Peer Competition | .239** | | .102** |
|  | Peer Cooperation | .306** | | .133** |
|  | Fear of Failure | .128** | | .128** |
|  | Work Mastery | .439** | | - |
|  | Gender | -.018 | | - |
|  | Grade | - | | - |
|  | ESCS | .075** | | - |
|  | School Type | - | | .015 |
|  | School Size | - | | .028* |
| 398Kazakhstan (*n*_student_ = 19507, *n*_school_ = 616) | | | | |
|  | Peer Competition | .150** | | .084** |
|  | Peer Cooperation | .318** | | .193** |
|  | Fear of Failure | -.022** | | - |
|  | Work Mastery | .325** | | - |
|  | Gender | -.112** | | - |
|  | Grade | .122** | | - |
|  | ESCS | .039** | | -.059** |
|  | School Type | - | | -.012 |
|  | School Size | - | | -.069** |
| 400Jordan (*n*_student_ = 8963, *n*_school_ = 313) | | | | |
|  | Peer Competition | .352** | | .190** |
|  | Peer Cooperation | .337** | | .159** |
|  | Fear of Failure | .069** | | - |
|  | Work Mastery | .500** | | - |
|  | Gender | -.168** | | - |
|  | Grade | .057** | | - |
|  | ESCS | .070** | | -.013 |
|  | School Type | - | | .011 |
|  | School Size | - | | .010 |
| 410Korea (*n*_student_ = 6650, *n*_school_ = 188) | | | | |
|  | Peer Competition | .127** | | .077** |
|  | Peer Cooperation | .277** | | .116** |
|  | Fear of Failure | -0.015 | | - |
|  | Work Mastery | .462** | | - |
|  | Gender | .045** | | - |
|  | Grade | 0.016 | | - |
|  | ESCS | .122** | | .049** |
|  | School Type | - | | -.049** |
|  | School Size | - | | -.005 |
| 428Latvia (*n*_student_ = 5303, *n*_school_ = 308) | | | | |
|  | Peer Competition | .184** | | .145** |
|  | Peer Cooperation | .205** | | .052** |
|  | Fear of Failure | .073** | | - |
|  | Work Mastery | .332** | | - |
|  | Gender | -.053** | | - |
|  | Grade | .015 | | - |
|  | ESCS | .112** | | .041** |
|  | School Type | - | | .000 |
|  | School Size | - | | -.027 |
| 440Lithuania (*n*_student_ = 6885, *n*_school_ = 362) | | | | |
|  | Peer Competition | .163** | | .133** |
|  | Peer Cooperation | .315** | | .170** |
|  | Fear of Failure | .082** | | - |
|  | Work Mastery | .423** | | - |
|  | Gender | -.129** | | - |
|  | Grade | -.013 | | - |
|  | ESCS | .133** | | .064** |
|  | School Type | - | | -.039** |
|  | School Size | - | | .038** |
| 442Luxembourg (*n*_student_ = 5230, *n*_school_ = 44) | | | | |
|  | Peer Competition | .161** | | .009 |
|  | Peer Cooperation | .231** | | .004 |
|  | Fear of Failure | .171** | | - |
|  | Work Mastery | .504** | | - |
|  | Gender | -.149** | | - |
|  | Grade | .032* | | - |
|  | ESCS | .071** | | .012 |
|  | School Type | - | | -.024 |
|  | School Size | - | | .007 |
| 446Macao (*n*_student_ = 3775, *n*_school_ = 45) | | | | |
|  | Peer Competition | .179** | | .094** |
|  | Peer Cooperation | .283** | | .108** |
|  | Fear of Failure | .078** | | - |
|  | Work Mastery | .390** | | - |
|  | Gender | -.115** | | - |
|  | Grade | .099** | | - |
|  | ESCS | .147** | | .061** |
|  | School Type | - | | -.029 |
|  | School Size | - | | .019 |
| 458Malaysia (*n*_student_ = 6111, *n*_school_ = 191) | | | | |
|  | Peer Competition | .351** | | .296** |
|  | Peer Cooperation | .423** | | .343** |
|  | Fear of Failure | .099** | | - |
|  | Work Mastery | .518** | | - |
|  | Gender | -.132** | | - |
|  | Grade | .164** | | - |
|  | ESCS | .092** | | .024 |
|  | School Type | - | | .080** |
|  | School Size | - | | -.173** |
| 470Malta (*n*_student_ = 3363, *n*_school_ = 50) | | | | |
|  | Peer Competition | .236** | | .099** |
|  | Peer Cooperation | .247** | | .101** |
|  | Fear of Failure | .085** | | - |
|  | Work Mastery | .478** | | - |
|  | Gender | -.094** | | - |
|  | Grade | .052** | | - |
|  | ESCS | .090** | | .044* |
|  | School Type | - | | -.039* |
|  | School Size | - | | .029 |
| 484Mexico (*n*_student_ = 7299, *n*_school_ = 286) | | | | |
|  | Peer Competition | .167** | | .043** |
|  | Peer Cooperation | .241** | | .085** |
|  | Fear of Failure | -.018 | | - |
|  | Work Mastery | .340** | | - |
|  | Gender | -.100** | | - |
|  | Grade | .093** | | - |
|  | ESCS | .050** | | .047** |
|  | School Type | - | | -.022 |
|  | School Size | - | | .014 |
| 498Moldova(*n*_student_ = 5367, *n*_school_ = 236) | | | | |
|  | Peer Competition | .207** | | .111** |
|  | Peer Cooperation | .352** | | .157** |
|  | Fear of Failure | .087** | | - |
|  | Work Mastery | .480** | | - |
|  | Gender | -.168** | | - |
|  | Grade | .019 | | - |
|  | ESCS | .128** | | -.014 |
|  | School Type | - | | -.017 |
|  | School Size | - | | -.046** |
| 499Montenegro (*n*_student_ = 6666, *n*_school_ = 61) | | | | |
|  | Peer Competition | .225** | | .073** |
|  | Peer Cooperation | .338** | | .107** |
|  | Fear of Failure | .010 | | - |
|  | Work Mastery | .402** | | - |
|  | Gender | -.181** | | - |
|  | Grade | .007 | | - |
|  | ESCS | .026* | | .006 |
|  | School Type | - | | -.006 |
|  | School Size | - | | -.025 |
| 504Morocco (*n*_student_ = 6814, *n*_school_ = 179) | | | | |
|  | Peer Competition | .278** | | .081** |
|  | Peer Cooperation | .222** | | .074** |
|  | Fear of Failure | .033* | | - |
|  | Work Mastery | .330** | | - |
|  | Gender | -.093** | | - |
|  | Grade | .073** | | - |
|  | ESCS | .043** | | .031* |
|  | School Type | - | | -.040** |
|  | School Size | - | | .012 |
| 528Netherlands (*n*_student_ = 4765, *n*_school_ = 156) | | | | |
|  | Peer Competition | .115** | | .055** |
|  | Peer Cooperation | .184** | | .028 |
|  | Fear of Failure | .055** | | - |
|  | Work Mastery | .406** | | - |
|  | Gender | -.096** | | - |
|  | Grade | -.026 | | - |
|  | ESCS | -.018 | | -.058** |
|  | School Type | - | | .035* |
|  | School Size | - | | -.030 |
| 554New Zealand (*n*_student_ = 6173, *n*_school_ = 192) | | | | |
|  | Peer Competition | .170** | | .039** |
|  | Peer Cooperation | .288** | | .115** |
|  | Fear of Failure | .143** | | - |
|  | Work Mastery | .531** | | - |
|  | Gender | -.105** | | - |
|  | Grade | -0.018 | | - |
|  | ESCS | .158** | | .077** |
|  | School Type | - | | -.063** |
|  | School Size | - | | - |
| 591Panama (*n*_student_ = 6270, *n*_school_ = 253) | | | | |
|  | Peer Competition | .199** | | .046** |
|  | Peer Cooperation | .265** | | .049** |
|  | Fear of Failure | .012 | | - |
|  | Work Mastery | .331** | | - |
|  | Gender | -.069** | | - |
|  | Grade | .045** | | - |
|  | ESCS | -.036* | | -.085** |
|  | School Type | - | | .096** |
|  | School Size | - | | .021 |
| 604Peru (*n*_student_ = 6086, *n*_school_ = 340) | | | | |
|  | Peer Competition | .174** | | .058** |
|  | Peer Cooperation | .235** | | .066** |
|  | Fear of Failure | -.038* | | - |
|  | Work Mastery | .340** | | - |
|  | Gender | -.068** | | - |
|  | Grade | .073** | | - |
|  | ESCS | -.010 | | -.040** |
|  | School Type | - | | .046** |
|  | School Size | - | | .028 |
| 608Philippines (*n*_student_ = 7233, *n*_school_ = 187) | | | | |
|  | Peer Competition | .334** | | .146** |
|  | Peer Cooperation | .435** | | .176** |
|  | Fear of Failure | .161** | | - |
|  | Work Mastery | .450** | | - |
|  | Gender | -.158** | | - |
|  | Grade | .258** | | - |
|  | ESCS | .150** | | .113** |
|  | School Type | - | | -.027* |
|  | School Size | - | | .058** |
| 616Poland (*n*_student_ = 5625, *n*_school_ = 240) | | | | |
|  | Peer Competition | .153** | | .040** |
|  | Peer Cooperation | .207** | | .057** |
|  | Fear of Failure | .152** | | - |
|  | Work Mastery | .396** | | - |
|  | Gender | -.162** | | - |
|  | Grade | 0.026 | | - |
|  | ESCS | .110** | | .054** |
|  | School Type | - | | -.020 |
|  | School Size | - | | .001 |
| 620Portugal (*n*_student_ = 5932, *n*_school_ = 276) | | | | |
|  | Peer Competition | .133** | | .042** |
|  | Peer Cooperation | .257** | | .111** |
|  | Fear of Failure | .074** | | - |
|  | Work Mastery | .428** | | - |
|  | Gender | -.119** | | - |
|  | Grade | -.035** | | - |
|  | ESCS | .151** | | .082** |
|  | School Type | - | | -.050** |
|  | School Size | - | | -.005 |
| 634Qatar (*n*_student_ = 13828, *n*_school_ = 188) | | | | |
|  | Peer Competition | .310** | | .023* |
|  | Peer Cooperation | .330** | | .100** |
|  | Fear of Failure | .063** | | - |
|  | Work Mastery | .459** | | - |
|  | Gender | -.130** | | - |
|  | Grade | .073** | | - |
|  | ESCS | .103** | | .048** |
|  | School Type | - | | .020* |
|  | School Size | - | | -.049** |
| 642Romania (*n*_student_ = 5075, *n*_school_ = 170) | | | | |
|  | Peer Competition | .174** | | .127** |
|  | Peer Cooperation | .302** | | .149** |
|  | Fear of Failure | .073** | | - |
|  | Work Mastery | .401** | | - |
|  | Gender | -.232** | | - |
|  | Grade | -.014 | | - |
|  | ESCS | .071** | | .062** |
|  | School Type | - | | .021 |
|  | School Size | - | | .081** |
| 643Russian Federation (*n*_student_ = 7608, *n*_school_ = 263) | | | | |
|  | Peer Competition | .197** | | .070** |
|  | Peer Cooperation | .282** | | .115** |
|  | Fear of Failure | .042** | | - |
|  | Work Mastery | .451** | | - |
|  | Gender | -.008 | | - |
|  | Grade | .015 | | - |
|  | ESCS | .049** | | -.086** |
|  | School Type | - | | -.005 |
|  | School Size | - | | -.039** |
| 682Saudi Arabia (*n*_student_ = 6136, *n*_school_ = 234) | | | | |
|  | Peer Competition | .345** | | .144** |
|  | Peer Cooperation | .350** | | .160** |
|  | Fear of Failure | .067** | | - |
|  | Work Mastery | .498** | | - |
|  | Gender | -.097** | | - |
|  | Grade | .045** | | - |
|  | ESCS | .111** | | .063** |
|  | School Type | - | | .018 |
|  | School Size | - | | .003 |
| 688Serbia (*n*_student_ = 6609, *n*_school_ = 187) | | | | |
|  | Peer Competition | .187** | | .119** |
|  | Peer Cooperation | .287** | | .138** |
|  | Fear of Failure | .081** | | - |
|  | Work Mastery | .437** | | - |
|  | Gender | -.114** | | - |
|  | Grade | -.004 | | - |
|  | ESCS | .054** | | .051** |
|  | School Type | - | | -.008 |
|  | School Size | - | | .011 |
| 702Singapore (*n*_student_ = 6676, *n*_school_ = 166) | | | | |
|  | Peer Competition | .204** | | .055** |
|  | Peer Cooperation | .262** | | .086** |
|  | Fear of Failure | .128** | | - |
|  | Work Mastery | .468** | | - |
|  | Gender | -.088** | | - |
|  | Grade | .018 | | - |
|  | ESCS | .089** | | .051** |
|  | School Type | - | | - |
|  | School Size | - | | .013 |
| 703Slovak Republic (*n*_student_ = 5965, *n*_school_ = 376) | | | | |
|  | Peer Competition | .169** | | .084** |
|  | Peer Cooperation | .246** | | .151** |
|  | Fear of Failure | .107** | | - |
|  | Work Mastery | .377** | | - |
|  | Gender | -.089** | | - |
|  | Grade | .087** | | - |
|  | ESCS | .152** | | .130** |
|  | School Type | - | | -.021 |
|  | School Size | - | | .045** |
| 704Vietnam (*n*_student_ = 5377, *n*_school_ = 151) | | | | |
|  | Peer Competition | .020 | | .022 |
|  | Peer Cooperation | -.254** | | -.134** |
|  | Fear of Failure | .069** | | - |
|  | Work Mastery | -.242** | | - |
|  | Gender | .091** | | - |
|  | Grade | -.004 | | - |
|  | ESCS | .041** | | .090** |
|  | School Type | - | | -.034* |
|  | School Size | - | | .055** |
| 705Slovenia (*n*_student_ = 6401, *n*_school_ = 345) | | | | |
|  | Peer Competition | .171** | | .073** |
|  | Peer Cooperation | .284** | | .112** |
|  | Fear of Failure | .047** | | - |
|  | Work Mastery | .392** | | - |
|  | Gender | -.017 | | - |
|  | Grade | .000 | | - |
|  | ESCS | .062** | | .035** |
|  | School Type | - | | -.033* |
|  | School Size | - | | -.004 |
| 724Spain (*n*_student_ = 35943, *n*_school_ = 1089) | | | | |
|  | Peer Competition | .125** | | .055** |
|  | Peer Cooperation | .240** | | .087** |
|  | Fear of Failure | .056** | | - |
|  | Work Mastery | .448** | | - |
|  | Gender | -.096** | | - |
|  | Grade | .158** | | - |
|  | ESCS | .147** | | .056** |
|  | School Type | - | | -.054** |
|  | School Size | - | | .020** |
| 752Sweden (*n*_student_ = 5504, *n*_school_ = 223) | | | | |
|  | Peer Competition | .093** | | .067** |
|  | Peer Cooperation | .234** | | .121** |
|  | Fear of Failure | .048** | | - |
|  | Work Mastery | .550** | | - |
|  | Gender | -.022 | | - |
|  | Grade | .006 | | - |
|  | ESCS | .110** | | .015 |
|  | School Type | - | | - |
|  | School Size | - | | .065** |
| 756Switzerland (*n*_student_ = 5822, *n*_school_ = 228) | | | | |
|  | Peer Competition | .156** | | .069** |
|  | Peer Cooperation | .177** | | .037** |
|  | Fear of Failure | .135** | | - |
|  | Work Mastery | .459** | | - |
|  | Gender | -.121** | | - |
|  | Grade | .093** | | - |
|  | ESCS | .050** | | .044** |
|  | School Type | - | | -.012 |
|  | School Size | - | | .048** |
| 764Thailand (*n*_student_ = 8633, *n*_school_ = 290) | | | | |
|  | Peer Competition | .207** | | .131** |
|  | Peer Cooperation | .352** | | .198** |
|  | Fear of Failure | .047** | | - |
|  | Work Mastery | .464** | | - |
|  | Gender | -.132** | | - |
|  | Grade | .083** | | - |
|  | ESCS | .095** | | .095** |
|  | School Type | - | | .027* |
|  | School Size | - | | .005 |
| 784United Arab Emirates (*n*_student_ = 19277, *n*_school_ = 755) | | | | |
|  | Peer Competition | .301** | | .123** |
|  | Peer Cooperation | .334** | | .157** |
|  | Fear of Failure | .070** | | - |
|  | Work Mastery | .479** | | - |
|  | Gender | -.118** | | - |
|  | Grade | .046** | | - |
|  | ESCS | .068** | | -.006 |
|  | School Type | - | | .034** |
|  | School Size | - | | .010 |
| 792Turkey (*n*_student_ = 6890, *n*_school_ = 186) | | | | |
|  | Peer Competition | .199** | | .028* |
|  | Peer Cooperation | .215** | | .047** |
|  | Fear of Failure | .112** | | - |
|  | Work Mastery | .405** | | - |
|  | Gender | -.079** | | - |
|  | Grade | .011 | | - |
|  | ESCS | -.039** | | -.065** |
|  | School Type | - | | -.026* |
|  | School Size | - | | -.016 |
| 804Ukraine (*n*_student_ = 5998, *n*_school_ = 250) | | | | |
|  | Peer Competition | .145** | | .052** |
|  | Peer Cooperation | .325** | | .130** |
|  | Fear of Failure | .035** | | - |
|  | Work Mastery | .326** | | - |
|  | Gender | -.086** | | - |
|  | Grade | .075** | | - |
|  | ESCS | .102** | | .015 |
|  | School Type | - | | -.021 |
|  | School Size | - | | .008 |
| 826United Kingdom (*n*_student_ = 13818, *n*_school_ = 471) | | | | |
|  | Peer Competition | .168** | | .052** |
|  | Peer Cooperation | .267** | | .074** |
|  | Fear of Failure | .068** | | - |
|  | Work Mastery | .472** | | - |
|  | Gender | -.081** | | - |
|  | Grade | .019* | | - |
|  | ESCS | .125** | | -.003 |
|  | School Type | - | | -.013 |
|  | School Size | - | | .018 |
| 840United States (*n*_student_ = 4838, *n*_school_ = 164) | | | | |
|  | Peer Competition | .146** | | .018 |
|  | Peer Cooperation | .277** | | .066** |
|  | Fear of Failure | .032* | | - |
|  | Work Mastery | .413** | | - |
|  | Gender | -.174** | | - |
|  | Grade | -.017 | | - |
|  | ESCS | .054** | | -.048** |
|  | School Type | - | | -.017 |
|  | School Size | - | | -.016 |
| 858Uruguay (*n*_student_ = 5263, *n*_school_ = 189) | | | | |
|  | Peer Competition | .120** | | .039* |
|  | Peer Cooperation | .223** | | .066** |
|  | Fear of Failure | .085** | | - |
|  | Work Mastery | .355** | | - |
|  | Gender | -.075** | | - |
|  | Grade | .089** | | - |
|  | ESCS | .066** | | .018 |
|  | School Type | - | | -.007 |
|  | School Size | - | | .014 |
| 975B-S-J-Z (China) (*n*_student_ = 12058, *n*_school_ = 361) | | | | |
|  | Peer Competition | .162** | | .067** |
|  | Peer Cooperation | .347** | | .177** |
|  | Fear of Failure | -.053** | | - |
|  | Work Mastery | .354** | | - |
|  | Gender | -.023* | | - |
|  | Grade | -.020* | | - |
|  | ESCS | .154** | | .154** |
|  | School Type | - | | .012 |
|  | School Size | - | | -.029** |
| 982Moscow Region (RUS) (*n*_student_ = 2016, *n*_school_ = 61) | | | | |
|  | Peer Competition | .164** | | .042 |
|  | Peer Cooperation | .284** | | .124** |
|  | Fear of Failure | .062** | | - |
|  | Work Mastery | .440** | | - |
|  | Gender | .026 | | - |
|  | Grade | -.024 | | - |
|  | ESCS | .091** | | -.032 |
|  | School Type | - | | - |
|  | School Size | - | | -.029 |
| 983Tatarstan (RUS) (*n*_student_ = 5816, *n*_school_ = 239) | | | | |
|  | Peer Competition | | .193** | .063** |
|  | Peer Cooperation | | .283** | .100** |
|  | Fear of Failure | | .028* | - |
|  | Work Mastery | | .415** | - |
|  | Gender | | -.003 | - |
|  | Grade | | -.010 | - |
|  | ESCS | | .061** | -.034* |
|  | School Type | | - | - |
|  | School Size | | - | -.045** |

***Notes.*** **p* < .05, ***p* < .01; *n*_student_ = number of students; *n*_school_ = number of schools; ESCS = socioeconomic status.

**Table S3**

*Multilevel Structural Equation Modeling of the Effects of Peer Cooperation and Competition on Mastery-approach Goals across Countries*

|  |  | Mastery-approach Goals | | |  |  |  |
| --- | --- | --- | --- | --- | --- | --- | --- |
|  |  | Individual level | | School level |  |  |  |
| 8Albania (*n*_student_ = 6359, *n*_school_ = 327) | | | | |  |  |  |
|  | Peer Competition | .186*** | | .163 |  |  |  |
|  | Peer Cooperation | .289*** | | .453** |  |  |  |
| 31Baku (Azerbaijan) (*n*_student_ = 6827, *n*_school_ = 197) | | | | |  |  |  |
|  | Peer Competition | .191*** | | .024 |  |  |  |
|  | Peer Cooperation | .222*** | | .423** |  |  |  |
| 32Argentina (*n*_student_ = 11975, *n*_school_ = 455) | | | | |  |  |  |
|  | Peer Competition | .125*** | | -.001 |  |  |  |
|  | Peer Cooperation | .248*** | | .024 |  |  |  |
| 36Australia (*n*_student_ = 14273, *n*_school_ = 763) | | | | |  |  |  |
|  | Peer Competition | .136*** | | .112 |  |  |  |
|  | Peer Cooperation | .253*** | | .195* |  |  |  |
| 40Austria (*n*_student_ = 6802, *n*_school_ = 291) | | | | |  |  |  |
|  | Peer Competition | .112*** | | .239 |  |  |  |
|  | Peer Cooperation | .199*** | | .463*** |  |  |  |
| 56Belgium (*n*_student_ = 8475, *n*_school_ = 288) | | | | |  |  |  |
|  | Peer Competition | .081*** | | -.388*** |  |  |  |
|  | Peer Cooperation | .183*** | | -.498*** |  |  |  |
| 70Bosnia and Herzegovina (*n*_student_ = 6480, *n*_school_ = 213) | | | | |  |  |  |
|  | Peer Competition | .165*** | | .483** |  |  |  |
|  | Peer Cooperation | .227*** | | .424* |  |  |  |
| 76Brazil (*n*_student_ = 10691, *n*_school_ = 597) | | | | |  |  |  |
|  | Peer Competition | .103*** | | .311*** |  |  |  |
|  | Peer Cooperation | .179*** | | -.090 |  |  |  |
| 96Brunei Darussalam (*n*_student_ = 6828, *n*_school_ = 55) | | | | |  |  |  |
|  | Peer Competition | .214*** | | .013 |  |  |  |
|  | Peer Cooperation | .192*** | | .109 |  |  |  |
| 100Bulgaria (*n*_student_ = 5294, *n*_school_ = 197) | | | | |  |  |  |
|  | Peer Competition | .168*** | | -.170 |  |  |  |
|  | Peer Cooperation | .239*** | | .365 |  |  |  |
| 112Belarus (*n*_student_ = 5803, *n*_school_ = 234) | | | | |  |  |  |
|  | Peer Competition | .101*** | | .161 |  |  |  |
|  | Peer Cooperation | .220*** | | .313* |  |  |  |
| 152Chile (*n*_student_ = 7621, *n*_school_ = 254) | | | | |  |  |  |
|  | Peer Competition | .106*** | | -.228 |  |  |  |
|  | Peer Cooperation | .221*** | | .027 |  |  |  |
| 158Chinese Taipei (*n*_student_ = 7243, *n*_school_ = 192) | | | | |  |  |  |
|  | Peer Competition | .156*** | | .177 |  |  |  |
|  | Peer Cooperation | .227*** | | .368** |  |  |  |
| 170Colombia (*n*_student_ = 7522, *n*_school_ = 247) | | | | |  |  |  |
|  | Peer Competition | .124*** | | .087 |  |  |  |
|  | Peer Cooperation | .182*** | | -.015 |  |  |  |
| 188Costa Rica (*n*_student_ = 7221, *n*_school_ = 205) | | | | |  |  |  |
|  | Peer Competition | .128*** | | -.278* |  |  |  |
|  | Peer Cooperation | .223*** | | -.056 |  |  |  |
| 191Croatia (*n*_student_ = 6609, *n*_school_ = 183) | | | | |  |  |  |
|  | Peer Competition | .024 | | -.080 |  |  |  |
|  | Peer Cooperation | .274*** | | .366** |  |  |  |
| 203Czech Republic (*n*_student_ = 7019, *n*_school_ = 333) | | | | |  |  |  |
|  | Peer Competition | .077*** | | -.174 |  |  |  |
|  | Peer Cooperation | .216*** | | .396** |  |  |  |
| 208Denmark (*n*_student_ = 7657, *n*_school_ = 348) | | | | |  |  |  |
|  | Peer Competition | .082*** | | .066 |  |  |  |
|  | Peer Cooperation | .260*** | | .152 |  |  |  |
| 214Dominican Republic (*n*_student_ = 5674, *n*_school_ = 235) | | | | |  |  |  |
|  | Peer Competition | .192*** | | .199 |  |  |  |
|  | Peer Cooperation | .225*** | | .407*** |  |  |  |
| 233Estonia (*n*_student_ = 5316, *n*_school_ = 230) | | | | |  |  |  |
|  | Peer Competition | .140*** | | .225* |  |  |  |
|  | Peer Cooperation | .239*** | | .364*** |  |  |  |
| 246Finland (*n*_student_ = 5649, *n*_school_ = 214) | | | | |  |  |  |
|  | Peer Competition | .123*** | | .315 |  |  |  |
|  | Peer Cooperation | .275*** | | .043 |  |  |  |
| 250France (*n*_student_ = 6308, *n*_school_ = 252) | | | | |  |  |  |
|  | Peer Competition | .098*** | | .191 |  |  |  |
|  | Peer Cooperation | .210*** | | .083 |  |  |  |
| 268Georgia (*n*_student_ = 5572, *n*_school_ = 321) | | | | |  |  |  |
|  | Peer Competition | .101*** | | -.286* |  |  |  |
|  | Peer Cooperation | .257*** | | .609*** |  |  |  |
| 276Germany (*n*_student_ = 5451, *n*_school_ = 223) | | | | |  |  |  |
|  | Peer Competition | .146*** | | .469* |  |  |  |
|  | Peer Cooperation | .214*** | | .041 |  |  |  |
| 300Greece (*n*_student_ = 6403, *n*_school_ = 242) | | | | |  |  |  |
|  | Peer Competition | .107*** | | .482*** |  |  |  |
|  | Peer Cooperation | .230*** | | .315* |  |  |  |
| 344Hong Kong (*n*_student_ = 6037, *n*_school_ = 152) | | | | |  |  |  |
|  | Peer Competition | .130*** | | .119 |  |  |  |
|  | Peer Cooperation | .263*** | | .351* |  |  |  |
| 348Hungary (*n*_student_ = 5132, *n*_school_ = 238) | | | | |  |  |  |
|  | Peer Competition | .110*** | | .282 |  |  |  |
|  | Peer Cooperation | .265*** | | .474** |  |  |  |
| 352Iceland (*n*_student_ = 3296, *n*_school_ = 142) | | | | |  |  |  |
|  | Peer Competition | .082*** | | .376 |  |  |  |
|  | Peer Cooperation | .285*** | | .269 |  |  |  |
| 360Indonesia (*n*_student_ = 12098, *n*_school_ = 397) | | | | |  |  |  |
|  | Peer Competition | .127*** | | -.218** |  |  |  |
|  | Peer Cooperation | .295*** | | .277*** |  |  |  |
| 372Ireland (*n*_student_ = 5577, *n*_school_ = 157) | | | | |  |  |  |
|  | Peer Competition | .111*** | | -.051 |  |  |  |
|  | Peer Cooperation | .236*** | | .409** |  |  |  |
| 380Italy (*n*_student_ = 11785, *n*_school_ = 542) | | | | |  |  |  |
|  | Peer Competition | .123*** | | .371*** |  |  |  |
|  | Peer Cooperation | .215*** | | -.121 |  |  |  |
| 383Kosovo (*n*_student_ = 5058, *n*_school_ = 211) | | | | |  |  |  |
|  | Peer Competition | .196*** | | -.070 |  |  |  |
|  | Peer Cooperation | .315*** | | .740*** |  |  |  |
| 392Japan (*n*_student_ = 6109, *n*_school_ = 183) | | | | |  |  |  |
|  | Peer Competition | .164*** | | -.016 |  |  |  |
|  | Peer Cooperation | .255*** | | .334* |  |  |  |
| 398Kazakhstan (*n*_student_ = 19507, *n*_school_ = 616) | | | | |  |  |  |
|  | Peer Competition | .092*** | | -.053 |  |  |  |
|  | Peer Cooperation | .261*** | | .598*** |  |  |  |
| 400Jordan (*n*_student_ = 8963, *n*_school_ = 313) | | | | |  |  |  |
|  | Peer Competition | .235*** | | .481*** |  |  |  |
|  | Peer Cooperation | .220*** | | .071 |  |  |  |
| 410Korea (*n*_student_ = 6650, *n*_school_ = 188) | | | | |  |  |  |
|  | Peer Competition | .164*** | | .326** |  |  |  |
|  | Peer Cooperation | .274*** | | .461*** |  |  |  |
| 428Latvia (*n*_student_ = 5303, *n*_school_ = 308) | | | | |  |  |  |
|  | Peer Competition | .131*** | | .361*** |  |  |  |
|  | Peer Cooperation | .196*** | | -.043 |  |  |  |
| 440Lithuania (*n*_student_ = 6885, *n*_school_ = 362) | | | | |  |  |  |
|  | Peer Competition | .100*** | | .338** |  |  |  |
|  | Peer Cooperation | .281*** | | .295** |  |  |  |
| 442Luxembourg (*n*_student_ = 5230, *n*_school_ = 44) | | | | |  |  |  |
|  | Peer Competition | .128*** | | -.422 |  |  |  |
|  | Peer Cooperation | .208*** | | -.692** |  |  |  |
| 446Macao (*n*_student_ = 3775, *n*_school_ = 45) | | | | |  |  |  |
|  | Peer Competition | .152*** | | .594* |  |  |  |
|  | Peer Cooperation | .262*** | | .185 |  |  |  |
| 458Malaysia (*n*_student_ = 6111, *n*_school_ = 191) | | | | |  |  |  |
|  | Peer Competition | .208*** | | .244*** |  |  |  |
|  | Peer Cooperation | .296*** | | .698*** |  |  |  |
| 470Malta (*n*_student_ = 3363, *n*_school_ = 50) | | | | |  |  |  |
|  | Peer Competition | .190*** | | .504** |  |  |  |
|  | Peer Cooperation | .207*** | | .314 |  |  |  |
| 484Mexico (*n*_student_ = 7299, *n*_school_ = 286) | | | | |  |  |  |
|  | Peer Competition | .115*** | | .149 |  |  |  |
|  | Peer Cooperation | .217*** | | .217 |  |  |  |
| 498Moldova(*n*_student_ = 5367, *n*_school_ = 236) | | | | |  |  |  |
|  | Peer Competition | .126*** | | .266** |  |  |  |
|  | Peer Cooperation | .318*** | | .216 |  |  |  |
| 499Montenegro (*n*_student_ = 6666, *n*_school_ = 61) | | | | |  |  |  |
|  | Peer Competition | .160*** | | -.026 |  |  |  |
|  | Peer Cooperation | .262*** | | .862*** |  |  |  |
| 504Morocco (*n*_student_ = 6814, *n*_school_ = 179) | | | | |  |  |  |
|  | Peer Competition | .151*** | | .285 |  |  |  |
|  | Peer Cooperation | .197*** | | .208 |  |  |  |
| 528Netherlands (*n*_student_ = 4765, *n*_school_ = 156) | | | | |  |  |  |
|  | Peer Competition | .112*** | | .423** |  |  |  |
|  | Peer Cooperation | .180*** | | -.358** |  |  |  |
| 554New Zealand (*n*_student_ = 6173, *n*_school_ = 192) | | | | |  |  |  |
|  | Peer Competition | .129*** | | -.011 |  |  |  |
|  | Peer Cooperation | .250*** | | .506*** |  |  |  |
| 591Panama (*n*_student_ = 6270, *n*_school_ = 253) | | | | |  |  |  |
|  | Peer Competition | .145*** | | .200 |  |  |  |
|  | Peer Cooperation | .213*** | | .160 |  |  |  |
| 604Peru (*n*_student_ = 6086, *n*_school_ = 340) | | | | |  |  |  |
|  | Peer Competition | .118*** | | .023 |  |  |  |
|  | Peer Cooperation | .217*** | | -.055 |  |  |  |
| 608Philippines (*n*_student_ = 7233, *n*_school_ = 187) | | | | |  |  |  |
|  | Peer Competition | .204*** | | .111 |  |  |  |
|  | Peer Cooperation | .327*** | | .472** |  |  |  |
| 616Poland (*n*_student_ = 5625, *n*_school_ = 240) | | | | |  |  |  |
|  | Peer Competition | .117*** | | .069 |  |  |  |
|  | Peer Cooperation | .188*** | | .098 |  |  |  |
| 620Portugal (*n*_student_ = 5932, *n*_school_ = 276) | | | | |  |  |  |
|  | Peer Competition | .107*** | | .231 |  |  |  |
|  | Peer Cooperation | .220*** | | .486** |  |  |  |
| 634Qatar (*n*_student_ = 13828, *n*_school_ = 188) | | | | |  |  |  |
|  | Peer Competition | .238*** | | -.434*** |  |  |  |
|  | Peer Cooperation | .248*** | | .516*** |  |  |  |
| 642Romania (*n*_student_ = 5075, *n*_school_ = 170) | | | | |  |  |  |
|  | Peer Competition | .098*** | | .316** |  |  |  |
|  | Peer Cooperation | .266*** | | .356** |  |  |  |
| 643Russian Federation (*n*_student_ = 7608, *n*_school_ = 263) | | | | |  |  |  |
|  | Peer Competition | .138*** | | .022 |  |  |  |
|  | Peer Cooperation | .233*** | | .488*** |  |  |  |
| 682Saudi Arabia (*n*_student_ = 6136, *n*_school_ = 234) | | | | |  |  |  |
|  | Peer Competition | .242*** | | .164 |  |  |  |
|  | Peer Cooperation | .238*** | | .311* |  |  |  |
| 688Serbia (*n*_student_ = 6609, *n*_school_ = 187) | | | | |  |  |  |
|  | Peer Competition | .115*** | | .468** |  |  |  |
|  | Peer Cooperation | .238*** | | .313* |  |  |  |
| 702Singapore (*n*_student_ = 6676, *n*_school_ = 166) | | | | |  |  |  |
|  | Peer Competition | .168*** | | -.092 |  |  |  |
|  | Peer Cooperation | .229*** | | .304 |  |  |  |
| 703Slovak Republic (*n*_student_ = 5965, *n*_school_ = 376) | | | | |  |  |  |
|  | Peer Competition | .111*** | | .151 |  |  |  |
|  | Peer Cooperation | .196*** | | .404*** |  |  |  |
| 704Vietnam (*n*_student_ = 5377, *n*_school_ = 151) | | | | |  |  |  |
|  | Peer Competition | .027 | | .106 |  |  |  |
|  | Peer Cooperation | -.233*** | | -.421** |  |  |  |
| 705Slovenia (*n*_student_ = 6401, *n*_school_ = 345) | | | | |  |  |  |
|  | Peer Competition | .125*** | | .385* |  |  |  |
|  | Peer Cooperation | .244*** | | .369** |  |  |  |
| 724Spain (*n*_student_ = 35943, *n*_school_ = 1089) | | | | |  |  |  |
|  | Peer Competition | .109*** | | .193*** |  |  |  |
|  | Peer Cooperation | .225*** | | .267*** |  |  |  |
| 752Sweden (*n*_student_ = 5504, *n*_school_ = 223) | | | | |  |  |  |
|  | Peer Competition | .077*** | | .185 |  |  |  |
|  | Peer Cooperation | .223*** | | .359** |  |  |  |
| 756Switzerland (*n*_student_ = 5822, *n*_school_ = 228) | | | | |  |  |  |
|  | Peer Competition | .132*** | | .124 |  |  |  |
|  | Peer Cooperation | .188*** | | -.022 |  |  |  |
| 764Thailand (*n*_student_ = 8633, *n*_school_ = 290) | | | | |  |  |  |
|  | Peer Competition | .132*** | | .049 |  |  |  |
|  | Peer Cooperation | .299*** | | .591*** |  |  |  |
| 784United Arab Emirates (*n*_student_ = 19277, *n*_school_ = 755) | | | | |  |  |  |
|  | Peer Competition | .212*** | | .000 |  |  |  |
|  | Peer Cooperation | .241*** | | .339*** |  |  |  |
| 792Turkey (*n*_student_ = 6890, *n*_school_ = 186) | | | | |  |  |  |
|  | Peer Competition | .171*** | | -.220 |  |  |  |
|  | Peer Cooperation | .177*** | | .091 |  |  |  |
| 804Ukraine (*n*_student_ = 5998, *n*_school_ = 250) | | | | |  |  |  |
|  | Peer Competition | .105*** | | .020 |  |  |  |
|  | Peer Cooperation | .285*** | | .390** |  |  |  |
| 826United Kingdom (*n*_student_ = 13818, *n*_school_ = 471) | | | | |  |  |  |
|  | Peer Competition | .130*** | | .015 |  |  |  |
|  | Peer Cooperation | .240*** | | .053 |  |  |  |
| 840United States (*n*_student_ = 4838, *n*_school_ = 164) | | | | |  |  |  |
|  | Peer Competition | .129*** | | -.144 |  |  |  |
|  | Peer Cooperation | .258*** | | -.035 |  |  |  |
| 858Uruguay (*n*_student_ = 5263, *n*_school_ = 189) | | | | |  |  |  |
|  | Peer Competition | .118*** | | -.108 |  |  |  |
|  | Peer Cooperation | .219*** | | .249 |  |  |  |
| 975B-S-J-Z (China) (*n*_student_ = 12058, *n*_school_ = 361) | | | | |  |  |  |
|  | Peer Competition | .121*** | | -.052 |  |  |  |
|  | Peer Cooperation | .312*** | | .507 |  |  |  |
| 982Moscow Region (RUS) (*n*_student_ = 2016, *n*_school_ = 61) | | | | |  |  |  |
|  | Peer Competition | .075** | | .127 |  |  |  |
|  | Peer Cooperation | .246*** | | .602*** |  |  |  |
| 983Tatarstan (RUS) (*n*_student_ = 5816, *n*_school_ = 239) | | | | |  |  |  |
|  | Peer Competition | | .124*** | .159 |  |  |  |
|  | Peer Cooperation | | .250*** | .281 |  |  |  |

***Notes.*** **p* < .05; ***p* < .01; ****p* < .001; *n*_student_ = number of students; *n*_school_ = number of schools.

**Table S4**

*Individualist and Collectivist Countries*

| **Individualist Countries** | |  | **Collectivist Countries** | |
| --- | --- | --- | --- | --- |
| Country | Individualism Index |  | Country | Individualism Index |
| Argentina | 46 |  | Brazil | 38 |
| Australia | 90 |  | Bulgaria | 30 |
| Austria | 55 |  | Chile | 23 |
| Belgium | 75 |  | Chinese Taipei | 17 |
| Czech Republic | 58 |  | Colombia | 13 |
| Denmark | 74 |  | Costa Rica | 15 |
| Estonia | 60 |  | Croatia | 33 |
| Finland | 63 |  | Greece | 35 |
| France | 71 |  | Hong Kong | 25 |
| Germany | 67 |  | Indonesia | 14 |
| Hungary | 80 |  | Jordan | 30 |
| Ireland | 70 |  | Korea | 18 |
| Italy | 76 |  | Malaysia | 26 |
| Japan | 46 |  | Mexico | 30 |
| Latvia | 70 |  | Panama | 11 |
| Lithuania | 60 |  | Peru | 16 |
| Luxembourg | 60 |  | Philippines | 32 |
| Malta | 59 |  | Portugal | 27 |
| Netherlands | 80 |  | Qatar | 25 |
| New Zealand | 79 |  | Romania | 30 |
| Poland | 60 |  | Russian Federation | 39 |
| Slovak Republic | 52 |  | Serbia | 25 |
| Spain | 51 |  | Singapore | 20 |
| Sweden | 71 |  | Vietnam | 10 |
| Switzerland | 68 |  | Slovenia | 27 |
| United Kingdom | 89 |  | Thailand | 20 |
| United States | 91 |  | Turkey | 37 |
|  |  |  | Uruguay | 36 |
|  |  |  | B-S-J-Z (China) | 20 |
|  |  |  | Moscow Region (RUS) | 39 |
|  |  |  | Tatarstan (RUS) | 39 |

***Note.*** A median split was conducted in terms of country scores on individualism. Those above the median were categorized as individualist and those below the median as collectivist countries.

**Part 2: Supplementary Figures**

**Figure S1**

*Multilevel Structural Equation Modeling of Peer Cooperation and Competition on Mastery-approach Goals with Random Slopes.*

***Notes*.** ^*^*p* < .05, ^***^ *p* < .001; ESCS = student socioeconomic status; School ESCS = school socioeconomic status; S1 refers to the random slope of individual perception of peer competition to mastery-approach goals, S2 refers to the random slope of individual perception of peer cooperation to mastery-approach goals, S3 refers to the random slope of school-level peer competition to mastery-approach goals, and S4 refers to the random slope of school-level peer cooperation to mastery-approach goals.

**Notes for Results of Figure S1**

The results indicated that significant random slopes of peer cooperation and competition at the school and country levels suggest that the magnitude of associations among individual-level peer cooperation and competition and mastery-approach goals differs as a function of membership in the school or country, or both. Relatedly, significant random slopes of school-level peer cooperation and competition at the country level suggest that the associations among individual-level peer cooperation and competition and mastery-approach goals differ as a function of country membership.

**Figure S2**

*Doubly-latent Multilevel Structural Equation Modeling among the Collectivist Countries.*

***Notes.*** ^*^*p* < .05, ^**^*p* < .01, ^***^*p* < .001; ESCS = student socioeconomic status; School ESCS = school socioeconomic status.

**Figure S3**

*Doubly-latent Multilevel Structural Equation Modeling among the Individualist Countries*

***Notes*.** ^*^*p* < .05, ^**^*p* < .01, ^***^*p* < .001; ESCS = student socioeconomic status; School ESCS = school socioeconomic status.

**Notes for cross-cultural comparison between individualist and collectivist countries**

Previous research has demonstrated that cultural values—particularly the distinction between collectivist and individualist countries —can shape the ways in which individuals interpret and respond to social and academic environments (Brewer & Chen. 2007; Triandis, 1989). In collectivist settings, where group harmony, shared responsibility, and loyalty to group norms are emphasized (Chen et al., 2006; Schwartz, 2013; King, 2022), peer competition can sometimes be perceived as a collective endeavor aimed at enhancing group outcomes (King et al., 2012), thereby potentially yielding a stronger positive association with mastery-approach goals.

Conversely, in individualist cultures, which prioritize personal autonomy and achievement (Fischer & Schwartz, 2011; Schwartz, 1999), objective individual success over mastery-based orientations might be prioritized. However, limited research has extended to a direct comparison of cultural differences in motivational processes. Given that prior research suggests cultural orientations influence motivational processes (Elliot et al., 2011; King & McInerney, 2016), it is crucial to assess the structural equivalence of the proposed model in distinct cultural settings. Therefore, the current study also explore whether the associations between peer cooperation, competition, and mastery-approach goals are consistent across collectivist and individualist cultures.

**Method**

**Measures**

***Individualism index***

Countries were classified into two groups according to the median value of the individualism index (Hofstede et al, 2010). Nations with an individualism index above the median were designated as individualist countries, whereas those with an index below the median were classified as collectivist countries (see Table S4). Countries for which the individualism index was not available in Hofstede et al. (2010) were excluded from further analysis.

***Data analysis***

We used multilevel multigroup doubly latent ML-SEM in both collectivist and individualist countries, for a rigorous examination of whether the hypothesized associations hold across cultural contexts. A significant change in *χ*² (△*χ*²) indicates that the magnitude of path coefficients varies between cultural groups, allowing for the conclusion that structural differences exist across cultures (Asparouhov & Muthén, 2006).

**Results**

Both individual-level peer cooperation (collectivist countries: *β* = .13, *p* < .001, medium effect; individualist culture: *β* = .11, *p* < .001, medium effect) and competition (collectivist countries: *β* = .08, *p* < .001, small effect; individualist countries: *β* = .05, *p* < .001, small effect) are positively associated with mastery-approach goals (See Figure S2 and S3).

The differences emerged at the school level. Both school-level peer cooperation (*β* = .33, *p* < .001, large effect) and peer competition (*β* = .15, *p* < .05, medium effect) were positively associated with mastery-approach goals among collectivist countries; however, among individualist countries, only the positive association between school-level peer cooperation and mastery-approach goals was significant (*β* = .24, *p* < .05, medium effect).

Furthermore, results of the multi-group multi-level SEM revealed that there were cross-cultural differences in the associations between peer competition and mastery-approach goals. Notably, school-level peer competition related more with mastery-approach goals in the collectivist countries, with △*χ*^2^(4) = 13.644, *p* < .05, rather than individualistic countries. No other cross-cultural differences were found.

**Discussion**

The supplementary analyses indicate that individual-level peer cooperation and peer competition both display consistent patterns in their positive associations with mastery-approach goals across collectivist and individualist contexts. Specifically, individual-level peer cooperation is positively associated with mastery-approach goals in both cultures, suggesting that direct perceptions of mutual support and collaboration among classmates can universally bolster students’ motivation to master learning tasks (Elliot et al., 2011; Warburton, 2017; Wentzel & Skinner, 2022). In a similar vein, individual-level peer competition also demonstrates positive associations with mastery-approach goals in both contexts, implying that students may perceive competition primarily as a personal challenge to refine their abilities (Fülöp, 2004, 2022; Guo et al., 2022).

Where cultural differences emerge more distinctly is at the school level, specifically regarding school-level peer competition. In collectivist settings, school-level peer competition appears more strongly linked to mastery-approach goals than in individualist contexts, possibly because a collectively oriented school climate can frame competition as a shared endeavor that encourages communal growth (Brewer & Chen; 2007; Järvelä et al., 2010; Hornsey et al., 2006; King et al., 2012). In contrast, in countries that emphasize individual autonomy and personal achievement (Fischer & Schwartz, 2011; Schwartz, 1999), the role of school-level peer competition on students’ mastery-approach goals is relatively diminished. Even so, it is noteworthy that school-level peer cooperation remains positively associated with mastery-approach goals across both cultures, aligning with the idea that a cooperative atmosphere tends to support students’ motivation for learning (Brown et al., 2008; Roseth et al., 2008).

Taken as a whole, these findings reveal more similarities than differences across cultural contexts, especially at the individual level, where both individual-level peer cooperation and peer competition are consistently positively associated with mastery-approach goals. Nonetheless, the varying role of school-level peer competition underscores the importance of considering cultural values when interpreting and applying these results.

**Supplementary References**

Brewer, M. B., & Chen, Y. R. (2007). Where (Who) Are Collectives in Collectivism? Toward Conceptual Clarification of Individualism and Collectivism. *Psychological Review, 114*(1), 133–151. [https://doi.org/10.1037/0033-295X.114.1.133](https://psycnet.apa.org/doi/10.1037/0033-295X.114.1.133)

Chen, X., French, D. C., & Schneider, B. H. (2006). Culture and Peer Relationships. In X. Chen, D. C. French, & B. H. Schneider (Eds.), *Peer Relationships in Cultural Context* (pp. 3–20). Cambridge University Press. [https://doi.org/10.1017/CBO9780511499739.001](https://psycnet.apa.org/doi/10.1017/CBO9780511499739.001)

Fischer, R., & Schwartz, S. (2011). Whence differences in value priorities? Individual, cultural, or artifactual sources. *Journal of Cross-Cultural Psychology*, *42*(7), 1127-1144. <https://doi.org/10.1177/0022022110381429>

Hornsey, M. J., Jetten, J., McAuliffe, B. J., & Hogg, M. A. (2006). The impact of individualist and collectivist group norms on evaluations of dissenting group members. *Journal of Experimental Social Psychology*, *42*(1), 57-68. <https://doi.org/10.1016/j.jesp.2005.01.006>

Järvelä, S., Volet, S., & Järvenoja, H. (2010). Research on motivation in collaborative learning: Moving beyond the cognitive–situative divide and combining individual and social processes. *Educational Psychologist*, *45*(1), 15-27. <https://doi.org/10.1080/00461520903433539>

Schwartz, S. H. (1999). A theory of cultural values and some implications for work. *Applied Psychology: An International Review*, *48*(1), 23–47. [https://doi.org/10.1111/j.1464-0597.1999.tb00047.x](https://psycnet.apa.org/doi/10.1111/j.1464-0597.1999.tb00047.x)

Schwartz, S. H. (2013). Culture matters: National value cultures, sources, and consequences. In R. S. Wyer, C.-y. Chiu, & Y.-y. Hong (Eds.), *Understanding Culture: Theory, Research, and Application* (pp. 127–150). Psychology Press.

Triandis, H. C. (1989). The self and social behavior in differing cultural contexts. *Psychological Review*, *96*(3), 506-520. <https://doi.org/10.1037/0033-295X.96.3.506>
